# The effects of contextual diversity on lexical processing: A scoping review

**DOI:** 10.3758/s13423-025-02761-y

**Published:** 2025-10-08

**Authors:** Rebecca Norman, J. S. H. Taylor, Jennifer M. Rodd

**Affiliations:** 1 Department of Language and Cognition, Division of Psychology and Language Sciences, University College London, Chandler House, 2 Wakefield Street, London, WC1N 1PF UK; 2 Department of Experimental Psychology, Division of Psychology and Language Sciences, University College London, 26 Bedford Way, London, WC1H 0AP UK

**Keywords:** Contextual diversity, Semantic diversity, Lexical processing, Contextual variation

## Abstract

Research into the effects of contextual diversity on lexical processing has flourished in the past 20 years, encompassing different tasks, populations, and languages, and informing influential theories of word learning. This review provides a comprehensive synthesis of the field. Eighty-six articles (145 experiments) composed of three distinct study types (behavioural [*N* = 111], computational modelling [*N* = 20], and corpus validations [*N* = 14]) met preregistered inclusion criteria. Across experiments, the terminology used for different diversity metrics has been inconsistently applied. We classify all metrics into four categories (count-based, computational, composite, unspecified) to standardise comparisons. Four key findings emerge from this review: Experiments that assessed the impact of diversity on *word-form* processing (*N* = 85) show a consistent high-diversity advantage, possibly because high-diversity words are more likely to be ‘needed’ in the future. Effects of diversity on *word-meaning* processing (*N* = 13) were more mixed, showing both low- and high-diversity benefits. We attribute these inconsistencies to varying task demands. Specifically, we conclude that selecting highly precise semantic information can be challenging for words that occur in variable contexts. Computational modelling studies indicate that diversity metrics that quantify the distinctiveness of contexts in which words occur better predict behaviour than simple context counts. Corpus validations show that diversity effects are consistent across languages. This review confirms that diversity in linguistic experience is a key organizational principle of the lexicon but indicates that current theories lack specificity when describing the underlying mechanisms. We make specific recommendations for future research within a structured research cycle.

## Introduction

Word frequency has long been shown to be an important determiner of lexical access, with high-frequency words being recognised faster and more accurately than low-frequency words (Monsell et al., 1989). This suggests that the principle of repetition is a core feature of lexical organisation (i.e., each encounter with a word increases its strength or accessibility in memory). Adelman et al. (2006) challenged this notion, suggesting that it is not the number of times a word is encountered that determines its strength in memory, but rather the number of distinct contexts in which it is experienced. They showed that ‘contextual diversity’, defined as the number of unique documents in which a word occurs, explained significantly more variance in lexical decision and word naming latencies than word frequency. Consequently, they theorised that the lexicon is organised according to ‘need’ (Anderson & Milson, 1989) rather than repetition—words appearing in more contexts are more likely to be needed in a new context and are therefore more accessible in memory.

Others have suggested measures of diversity that account for semantic overlap between the documents in which a word appears (Hoffman et al., 2013; Jones et al., 2012). For example, the words ‘perjury’ and ‘horrific’ are both similar in terms of contextual diversity (.83 and.77, respectively; Brysbaert & New, 2009). However, ‘perjury’ is limited in its usage to discussions of legal proceedings and is considerably lower in semantic diversity then ‘horrific’ (1.14 and 1.68, respectively; Hoffman et al., 2013), which can be used in a variety of contexts to describe something unpleasant. Semantic diversity accounts for unique variance in lexical decision and word naming performance, over and above both word frequency and Adelman et al.’s (2006) contextual diversity metric. This suggests that ‘need’ may be determined by the nature of the semantic contexts in which a word occurs. Words appearing in multiple semantic contexts are more broadly used and therefore more likely to be needed in a new context.

Since the publication of Adelman et al. (2006), the research interest in contextual diversity has drastically increased (Fig. 2). There is now a substantial body of work investigating the effects of diversity on processing of word forms (e.g., Adelman et al., 2006; Jones et al., 2012) and word meanings (e.g., Hoffman & Woollams, 2015) using a range of methodologies; for example, lexical decision (e.g., Adelman et al., 2006; Pexman et al., 2008), eye-tracking (e.g., Pagán et al., 2020), and computational modelling (e.g., Johns & Jones, 2022); on different populations, including children (e.g., Perea et al., 2013) and patients (e.g, Hoffman et al., 2011); and in several languages (e.g., Perea et al., 2013; Tsang & Zou, 2022).

In contrast to the proliferation of data concerning the effects of contextual diversity on lexical processing, comparatively little progress has been made in terms of the theory underlying contextual diversity effects. One major issue is that research is poorly integrated across subfields, with multiple theories emerging to explain related phenomena; for example, likely need (Adelman et al., 2006) for form processing and fan theory (Cook et al., 2006) for recognition memory. There is also a vast array of diversity metrics (Table 2), which have defined a ‘context’ in a multitude of different ways (Hoffman et al., 2013; Hollis, 2017; Johns et al., 2020). Despite the numerous metrics available, there are very few direct comparisons between them, so there is no clear picture as to why certain metrics explain some data better than others. Finally, where overarching theories do exist, they are relatively underspecified. For example, the ‘lexical quality’ (Perfetti & Hart, 2002) and ‘lexical legacy’ (Nation, 2017) hypotheses suggest that encountering words in varying linguistic environments leads to the development of stable word-form representations that are context independent (facilitating form processing) and more flexible meaning representations (facilitating use in new contexts). Though derived from cross-domain learning principles (see Raviv et al., 2022, for a review), these hypotheses do not specify the mechanisms by which diversity effects emerge and make only general predictions for empirical studies. These issues may reflect the wider ‘theory crisis’ in psychological research—that is, in attempting to solve the replication crisis, researchers have focused on hypothesis testing rather than advancing the theory underlying observed results (Muthukrishna & Henrich, 2019; Oberauer & Lewandowsky, 2019). With this in mind, we conducted a scoping review to produce a comprehensive map of the literature published to date that has investigated the effects of diversity and clearly identify how the results relate to theory, and what needs to be done to advance the field.

### Objectives

Our goal was to provide a holistic overview of contextual diversity research to date rather than to answer one specific research question. We therefore conducted a scoping review rather than a systematic review. Specifically, we aimed to identify higher order patterns of diversity effects across studies by summarising their methodologies, results, similarities, and differences. To achieve this, we developed seven review questions (RQs) to guide the data extraction process and subsequent synthesis of the findings:

1. How has diversity been defined in the literature? (i.e., what names have been given to the different metrics?)
2. How has diversity been operationalised across studies? (i.e., how has diversity been calculated?)
3. What outcome measures have been used to investigate the effects of diversity on word-form processing?
4. Does diversity affect word-form processing?
5. What outcome measures have been used to investigate the effects of diversity on word-meaning processing?
6. Does diversity affect word-meaning processing?
7. What factors identified in this review modulate the effects of contextual diversity?

In conducting this review, we identified several outcome measures that met our inclusion criteria but assessed aspects of lexical processing that could not be straightforwardly classified as either word-form or word-meaning processing. This led to the development of two research questions not included in the preregistration:

8. What other behavioural outcome measures have been used to investigate the effects of diversity on lexical processing?
9. Does diversity influence other behavioural outcomes?

In answering these questions, we will be able to determine where and why findings are consistent/inconsistent and identify key knowledge gaps. We will also be able to identify what stage in the research cycle each subfield is at and thus suggest useful avenues for future research.

## Methods

### Protocol and registration

The review protocol was developed in accordance with the JBI methodology for scoping reviews and the Preferred Reporting Items for Systematic Reviews and Meta-Analyses (PRISMA) extension for scoping reviews and was preregistered with the Open Science Framework on 05/03/2022 (https://osf.io/qmda2). Any changes to the preregistration are reported.

### Eligibility criteria

To be included in this review, studies had to meet the following criteria: Include a behavioural outcome measure that assessed how participants respond to familiar words that vary in some measure of diversity, clearly report how diversity has been defined, clearly report how diversity was manipulated, and report original findings. We did not impose any restrictions on age, language background, or whether participants had neurological impairments. There were no restrictions on the language or modality of the materials used in the studies or on date of publication. Studies were excluded if they were not available in English, were not included in a peer reviewed journal, or did not report original findings. In a change to the preregistered inclusion criteria, we also chose to exclude any training studies that tested the effect of diversity on word learning. This was due to the volume of studies returned and these will form a separate review.

### Information sources

To identify potentially relevant studies, the following databases were searched: Web of Science Core Collection, Scopus, PsycINFO, ERIC (Education Resources Information Center), and LLBA (Linguistics and Language Behaviour Abstracts). Database selection was developed with input from an experienced librarian. The electronic database searches were supplemented with scanning of reference lists of final included studies for additional relevant papers.

### Search strategy

To develop the search strategy, an initial search of Web of Science Core Collection was run on 08/02/2022 to identify key articles on the topic. Appropriate keywords were identified from the titles and abstracts of relevant articles and the index terms used to describe these articles. Using these keywords, a full search strategy was developed with input from an experienced librarian. This was validated by running the search on Web of Science Core Collection and seeing if it returned previously identified key studies. The initial searches were run on all databases using this strategy on 05/20/2022. Titles, abstracts, and keywords were searched. During title and abstract screening, additional keywords were identified and a second search strategy was developed incorporating these. A second round of searches was run against all databases on 06/16/2022 using this updated strategy. The final search strategy is documented in Table 1.

**Table 1:** Full search strategy used, databases searched, and date of search

Database	Date	Search strategy and keywords	
Web of Science Core Collection, SCOPUS, PsycINFO, ERIC, LLBA	16/06/2022	Titles, abstracts, keywords	“contextual diversity” OR “semantic diversity” OR “semantic distinctiveness” OR “document count” OR “context* varia*” OR “semantic varia*” OR “context* informativeness” AND word*

### Selection of sources of evidence

**Fig. 1:**
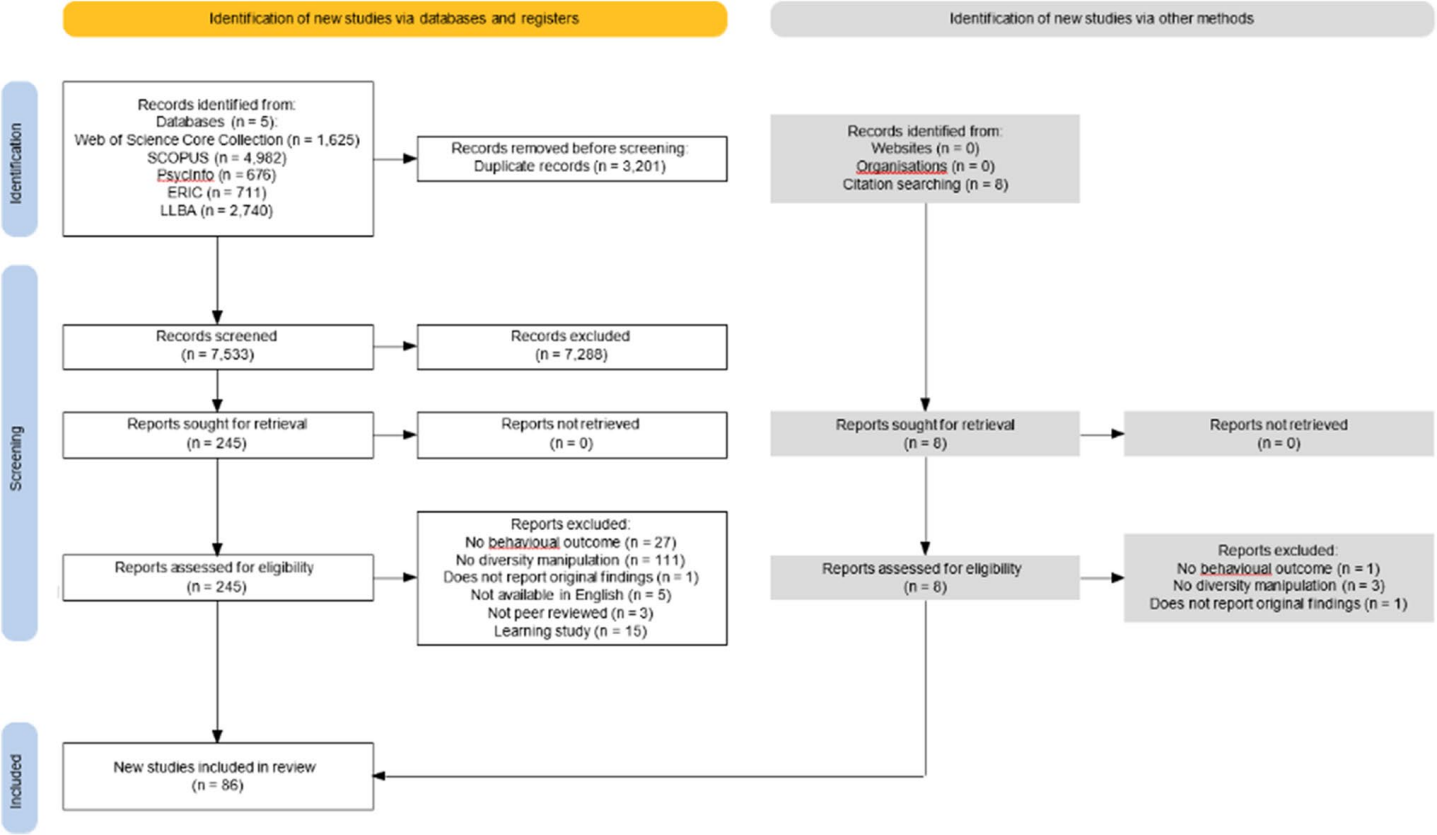
PRISMA flow diagram showing the article selection process

The results of the database searches were exported to EndNote 20 (EndNote Team, 2013) and duplicate articles were removed. The PRISMA flow diagram (Fig. 1) summarises the selection process. The resulting reference list was then exported to Rayyan (Ouzzani et al., 2016) and any additional duplicates were removed. Two reviewers independently screened the same 10% sample of the articles against the inclusion and exclusion criteria. Interrater agreement was high (92.86%). They discussed any disagreements, and the data extraction guidelines were amended for clarity. The remainder of the titles and abstracts were then screened and irrelevant articles removed. Agreement was high (96.95%) and disagreements were resolved through discussion. Following this, the full texts of all potentially relevant articles identified in the previous stage were screened by the two reviewers against the inclusion and exclusion criteria and irrelevant articles removed. Interrater agreement was again high (86.80%) and any disagreements were resolved through discussion. There were 86 articles in the final sample. A full list of the publications included in this review is provided in the supplementary materials on the OSF (https://osf.io/ktsfy).

### Data charting process

Data charting forms were developed in accordance with the JBI guidelines (Peters, 2017) using Microsoft Excel. The initial data charting form was piloted on previously identified key studies to determine what variables to extract. Full data charting was performed by one reviewer, with the second reviewer verifying the process by checking extraction for a subset of articles (10%). This initial data charting process revealed that where articles contained multiple experiments, extracting the data for each of these individually allowed for the most effective compilation of the findings. This gave a total of 145 experiments included in the final synthesis. Furthermore, subdividing these into different categories based on their common methodological approach was required to give a comprehensive overview of the field. As such, in a deviation from the preregistration, we devised three categories of experiment based on common methodologies and research aims. An experiment was not included in more than one category. Where an experiment could belong to more than one category, it was placed in the category that best fit the methodological approach and would allow for the most thorough data extraction. The three categories of experiment were as follows:

- 1. Behavioural studies.
  - These experiments all directly assess whether diversity influences behaviour. These studies investigate whether corpus-based diversity metrics, either preexisting or newly computed, have a relationship to behavioural measures (e.g., does high diversity lead to faster and/or more accurate responses in lexical decision). The experiments in this category form the largest part of the review and provide relevant information relating to all the research questions (RQs 1–9).
- 2. Computational modelling studies.
  - These studies set out to determine which diversity metrics perform best in explaining variance in behavioural data derived from megastudy datasets. These studies apply multiple different calculations of diversity metrics to the same datasets and explore how changing model parameters alters performance (e.g., do diversity metrics calculated over entire books explain more variance in behavioural data than metrics calculated over paragraphs). These studies are particularly relevant for our aim of reviewing how diversity has been defined and operationalised (RQ1, RQ2), but also provide information about whether diversity affects word-form processing (RQ4), and other behavioural outcome measures (RQ 9). In particular, they provide a more nuanced view on what type(s) of linguistic diversity are the most important determiners of performance on word processing tasks and provide additional information regarding the organisation of the lexicon.
- 3. Corpus validations.
  - These studies describe the construction of a number of different corpora across a range of different languages. Importantly, they report a number of lexical variables, including diversity metrics, that were extracted from these new corpora. These measures are then applied to behavioural data in order to validate the norms calculated and compared with norms extracted from other corpora. Unlike the other categories, these experiments do not explicitly set out to answer theoretical questions regarding the effects of diversity on behavioural performance, but nonetheless provide information about this. With respect to our review questions, these experiments provide information about the extent to which effects of diversity on word-form processing generalise across different languages and across corpora that use different types of linguistic information (e.g., subtitles vs websites and newspaper articles; RQ4, RQ7).

The original data charting forms were adapted for each of these methodological approaches. Throughout the data charting process, the forms were updated in an iterative manner when additional relevant variables were identified. The final data extraction forms with full documentation of the changes made to the preregistered forms are provided in the supplementary materials (https://osf.io/agszt).

### Data items

For each experiment, data were extracted regarding author name(s), year of publication, country where the study was conducted, source of behavioural data (new participants or existing datasets), population and sample size (in the case of new participants), source material (when diversity was calculated from a corpus or database), definition of diversity, definition of context, methodology (study design, operationalization of diversity, assessment of diversity, outcome measures), other variables included in the analysis/controlled for, results, and conclusions. The final database of included experiments including the data extracted for each is available on the OSF (https://osf.io/ys6ne).

### Synthesis of results

The results section will be broken down according to the three methodological categories set out above: (1) Behavioural studies, (2) Computational modelling studies, (3) Corpus validations.

Within each of these sections, the results will be organised into two parts. The first will cover how the operationalisation of diversity differs across experiments and the different terms that have been used to describe diversity metrics (RQs 1 & 2). The second will cover whether diversity influences word-form, meaning, and other aspects of lexical processing, and the factors that modulate these effects (e.g., the diversity metric used and where there are interactions between diversity and other item and participant level variables; RQs 3–9). Results are grouped according to tasks that use a similar method, which allows us to map the consistencies and inconsistences in methodologies and results.

We adopted a common data processing approach across this review. Where multiple metric types (e.g., contextual diversity and semantic diversity) and/or outcome measures (e.g., lexical decision and word naming) were used in a single experiment, the results for these were recorded separately. In addition, where multiple dependent measures (e.g., accuracy and reaction time) were used in a single experiment, the results have been recorded separately, as have results for experiments including two or more different groups (e.g., adults and children). Experiments where higher diversity resulted in a significant processing advantage are coded as ‘High-Diversity Advantage’, experiments where lower diversity resulted in a significant processing disadvantage are coded as ‘Low-Diversity Advantage’, and those where no significant effect was found are coded as ‘No Effect’. Experiments where a result was termed ‘marginally significant’ or ‘approaching significance’ (*p* >.05 or greater than researcher-specified significance level where experiments used a more restrictive criterion—e.g., correcting for multiple comparisons) were also coded as ‘No Effect’.

## Results

### Characteristics of sources of evidence

The results presented in this section were calculated from the combined data from all 145 experiments. The full descriptive statistics for all experiments included in this review are available in the supplementary materials (https://osf.io/rdfse).

#### Year of publication

**Fig. 2:**
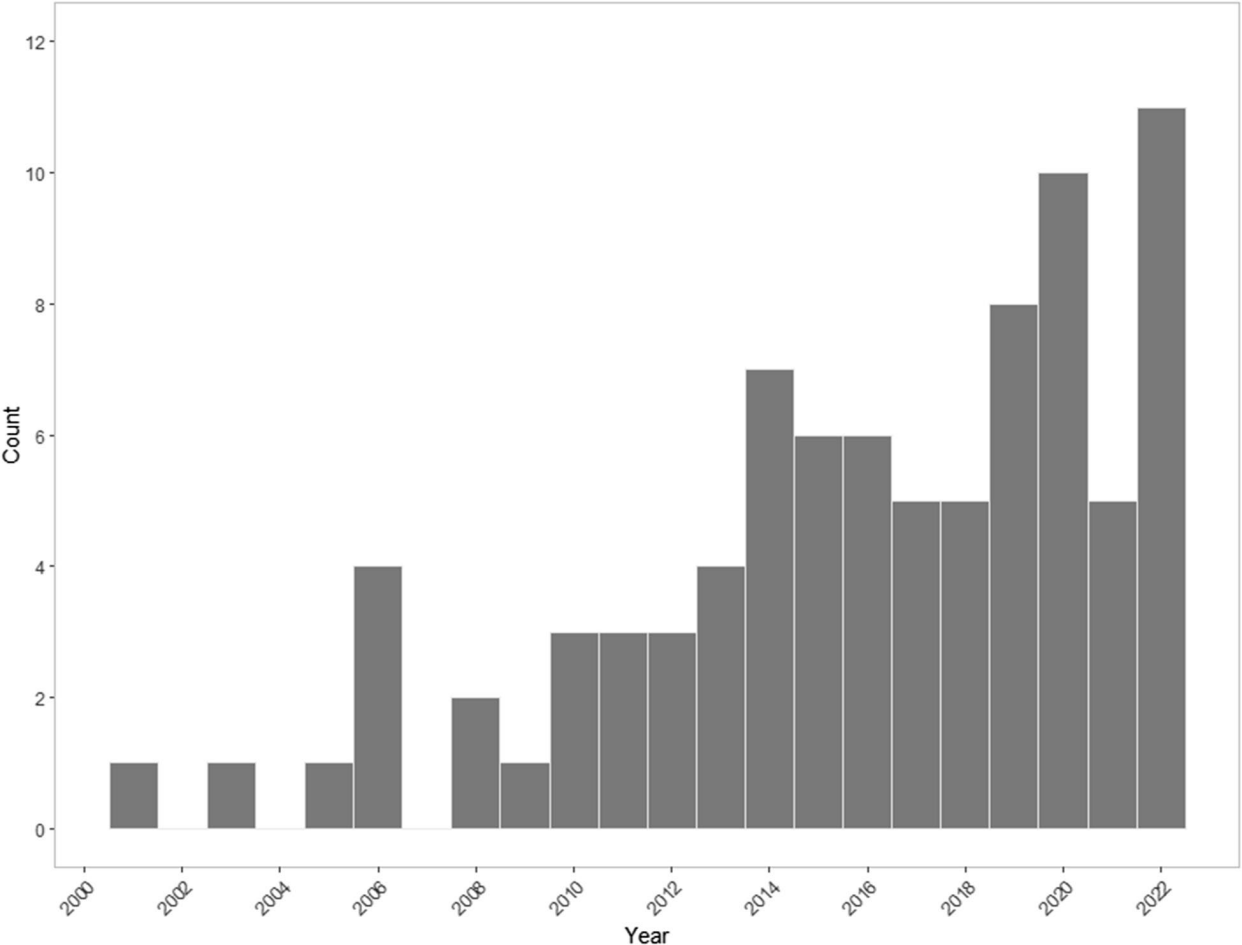
Total number of articles published investigating the effects of diversity on lexical processing

Research on the effects of diversity on lexical processing is a rapidly growing field, with an increase in the number of articles published after 2006 (Fig. 2).

#### Study design

Ninety-three experiments used primary data, 51 used secondary data, and one used a mix of both. Of the experiments conducted on primary data, 80 adopted a within-subject design, and 13 adopted a between-subjects design. Seventy-two experiments were conducted in person, 18 were conducted online, and three collected data both in person and online.

#### Population

Of the experiments that used primary data, 80 were conducted with adults, 10 were conducted with children, and two used both. In one case the population was not specified.

#### Country

Country is defined as the physical location in which participants completed the experiment. If this was not explicitly given, country of affiliation of the first author was used. Experiments from 18 different countries are included in this review. Two experiments were classified as multisite^1^, meaning that data were gathered in two or more different countries but the analyses were conducted on the pooled data. The majority of the experiments were conducted in the USA (40), Canada (34), and the UK (26). It is therefore not surprising that most were conducted in English (109); however, we identified experiments conducted in nine additional natural languages. Two experiments were conducted on stimuli from multiple languages but did not report the results as separate experiments. These were classified as mixed language.^2^

#### Diversity metrics

We identified 31 different diversity metrics that have been used. Table 2 gives details of these metrics, including the article in which they were first used, the original name of the metric, and their calculation. The 31 operationalisations can be grouped into four overarching categories (count, computational, composite, and unspecified) based on shared methodologies. It is clear that multiple different terms have been used to describe the same construct (e.g., ‘range’, ‘context variability’, and ‘contextual diversity’ are all calculated as a count of the documents in which a word appears).

**Table 2:** Details of the diversity metrics covered in this review

Category	Category definition	Author	Original name of diversity metric	Context size	Calculation of diversity
Count-based	Count of the contexts in a corpus in which a word appears without regard to the semantic content of those contexts	Adelman et al. (2006)	Contextual diversity	Entire document	Number of documents in which a word appears.
		Brysbaert and New (2009)	Contextual diversity	Subtitles for entire television show or film	Number of films or television shows in which a word appears.
		Hashimoto and Egbert (2019)	Range	Entire document	Number of documents in which a word appears.
		Hollis (2020)	CDrand	Entire document	Number of documents in which a word appears, with content words randomised.
		Hollis (2020)	Contextual diversity	Specified window size (2, 4, 8, 16, 32, 64, 128, 256, 512, 1024 words, or entire document)	Number of contexts in which a word appears.
		Johns et al. (2020)	Author prevalence	Entire works of an author	Number of authors who use a word.
		Johns et al. (2020)	Book prevalence	Entire book	Number of books in which a word occurs.
		Johns (2021b)	Discourse contextual diversity	Single subreddit	Number of subreddits (discourses) in which a word appears, within a reddit corpus.
		Johns (2021b)	User contextual diversity	Single comment	Number of comments in which a word appears, within a Reddit corpus.
		Johns and Jones (2022)	SDM_count	All comments produced by a user	Number of contexts in which a word appears that reach a specified SDM-derived diversity (Johns et al., 2020) threshold.
		Steyvers and Malmberg (2003)	Context variability	Entire document	Number of documents in which a word appears.
Computational	Apply computational procedures to corpora to derive diversity metrics that account for the semantic content of the contexts in which a word appears.				
Distributional	Compare the actual/observed distribution of words in a corpus to the expected distribution if words were distributed evenly throughout the corpus	Burch et al. (2016)	Dispersion	Single register category	Average of all pairwise comparisons between the frequency of occurrence of a word in any two register categories of the Corpus of Contemporary American English (COCA), divided by the average total frequency of occurrence the word across the corpus. Gives a value between 0 (all occurrences concentrated in one part of the corpus) and 1 (occurrences are distributed evenly).
		Heffernan et al. (2018)	Dispersion	Single speaker	Observed use of a word by one speaker within the Corpus of Kansai Vernacular Japanese compared with the expected usage (i.e., that each speaker produces an equal proportion of the words). The proportion of words produced by a speaker is calculated. For each word, the absolute values of the difference between the expected and observed proportions are summed and divided by 2, producing a value between 0 (used by one speaker) and 1 (evenly distributed across speakers).
		McDonald and Shillcock (2001)	Contextual distinctiveness	±5 words surrounding the target word (10-word window)	Comparison of the distribution of words surrounding the target word and the expected distribution of those words based on their frequencies of occurrence in the corpus. Contextual distinctiveness is the mean dissimilarity between these distributions. When this is large, a word appears in more specific topics and is high in contextual distinctiveness. A smaller difference, and low contextual distinctiveness, means a word is widely used across topics.^a^
LSA-derived	Latent semantic analysis (LSA) used to calculate diversity as a function of the mean distance between the contexts in which a word occurs	Cevoli et al. (2021)	Textual diversity	1,000-word section of text	Word-by-context co-occurrence matrix created (Hoffman et al., 2013). Scaled vectors for each context extracted using LSA by applying a log entropy weighting to the matrix to reduce its dimensionality via singular value decomposition. Cosine similarity between all pairs of contexts in which a word appears is calculated. Semantic diversity is the mean cosine similarity between all context vectors of a word.
		Chang and Lee (2018)	Semantic variability	150 words	LSA used to extract context vectors for a word. These are grouped into clusters based on mean cosine similarity using k-means clustering. Semantic variability is the mean cosine distance between context vectors within clusters, divided by the mean cosine distance between clusters.
		Hoffman et al. (2013)	Semantic diversity	1,000-word section of text	A word-by-context co-occurrence matrix logs the occurrence of each word in each context. Unscaled context vectors for each word extracted using LSA. Cosine similarity between all pairs of contexts in which a word appears is calculated. Semantic diversity is the mean pairwise cosine similarity between all a word’s context vectors.
Predict	Word embeddings (feature vectors representing a word’s meaning) created by predicting a word’s use over a corpus.	Hollis (2017)	Average need (continuous bag of words [CBOW])	Target word minus 5 words	Word embeddings are generated by presenting CBOW with a string of words and having it predict the next word in the sequence. Average need is derived by calculating the mean similarity of the target word embedding derived from CBOW and 1,000 random words, with the random words weighted by word frequency. Higher values indicate higher average need, or higher probability that the target word would be needed in a randomly selected context.
		Hollis (2017)	Average need (skip-gram)	Target word minus 5	Word embeddings are generated by presenting skip-gram with a target word and having it predict which words are present in the surrounding context. Average need is derived by calculating the mean similarity of the target word embedding derived from skip-gram and 1,000 random words, with the random words weighted by word frequency. Higher values indicate higher average need, or higher probability that the target word would be needed in a randomly selected context.
SDM-derived	Uses the semantic distinctiveness model (SDM; Johns et al., 2020; Jones et al., 2012) to compute diversity as a proportion of overlapping words across all contexts in which a word occurs.	Jones et al. (2012)	Semantic distinctiveness	Entire document	The SDM performs pairwise comparisons between all the contexts in which a word occurs and determines the proportion of overlapping words. A word’s semantic distinctiveness is the mean of these comparisons. Words higher in semantic distinctiveness appear in contexts with lower levels of semantic overlap.
		Johns et al. (2020)	Semantic distinctiveness	Entire document	In the updated version of the SDM, each word is initially represented by an empty vector, which is updated each time a word is encountered in a context. This results in a memory vector representing the meaning of the word. The SDM determines the strength with which the vector is updated based on the cosine similarity between the current context and the memory vector (all past contexts for that word). Lower levels of semantic overlap (derived from the proportion of overlapping words as in the original SDM) lead to greater updating of the semantic distinctiveness value and words higher in semantic distinctiveness appear in contexts with lower levels of semantic overlap.
		Johns et al. (2020)	Semantic diversity author prevalence	Entire works of an author	Uses the SDM (Johns et al., 2020) but the entire works of an author are taken as a context. Each time a word is used by a new author, the SDM updates the semantic distinctiveness value for that word based on the cosine similarity between the current context (author) and all other contexts (authors) that have used the word, stored in the memory vector.
		Johns et al. (2020)	Semantic diversity book prevalence	Entire book	Uses the SDM (Johns et al., 2020) but an entire book is taken as a context. Each time a word is used in a new book, the SDM updates the semantic distinctiveness value for that word based on the cosine similarity between the current context (book) and all other contexts (books) the word has been used in, stored in the memory vector.
		Johns (2021b)	Discourse semantic diversity (word representation)	Single subreddit	Uses the SDM (Johns et al., 2020) but a single subreddit (discourse) is taken as a context. Each time a word is used in a new subreddit, the SDM updates the semantic distinctiveness value for that word based on the cosine similarity between the current context (subreddit) and all other contexts (subreddits) the word has been used in, stored in the memory vector.
		Johns (2021b)	Discourse semantic diversity (population representation)	All comments produced by a user within a subreddit	Uses the SDM (Johns et al., 2020) but all the comments produced by a user within a subreddit (discourse) are taken as a context. When a word is used by a new user within a discourse, the SDM updates the semantic distinctiveness value for that word based on the cosine similarity between the current context (comments for that user within a specific subreddit) and all other contexts (comments from all previous users within a specific subreddit) the word has been used in, stored in the memory vector.
		Johns (2021b)	User semantic diversity (word representation)	Single comment	Uses the SDM (Johns et al., 2020) but a single comment on the reddit website is taken as a context. Each time a word is used in a new comment, the SDM updates the semantic distinctiveness value for that word based on the cosine similarity between the current context (single comment) and all other contexts (previous comments) the word has been used in, stored in the memory vector.
		Johns (2021b)	User semantic diversity (population representation)	All comments produced by a user within a subreddit	Uses the SDM (Johns et al., 2020) but all the comments produced by a user within a subreddit (discourse) are taken as a context. Each time a word is used in a new discourse by that user, the SDM updates the semantic distinctiveness value for that word based on the cosine similarity between the current context (all comments for that user within a specific subreddit) and all other contexts (previous comments from that user across subreddits) the word has been used in, stored in the memory vector.
Other		Hills et al. (2010)	Contextual diversity	Window sizes 2–100 words	Number of unique word types immediately occurring before and after a target word for a given window size. Words that cooccur with higher numbers of unique words are higher in contextual diversity.
		Hollis (2020)	Word burstiness	Specified window size (2, 4, 8, 16, 32, 64, 128, 256, 512, 1024 words or entire document)	A word’s frequency over the corpus divided by the number of contexts it appears in. Words that appear multiple times within the same context have a higher burstiness score.
Composite	Combine both count-based and computational metrics	Musz and Thompson-Schill (2015)	Semantic variability	Not given	Composite score created by *z*-scoring then averaging seven different diversity metrics. Composed of count-based metrics (Brysbaert & New, 2009; Hoffman et al., 2013), computational metrics (Hoffman et al., 2013), and topic modelling (Perea et al., 2013).
Unspecified	Calculation method not retrievable	Zeno et al. (1995)	Contextual dispersion	Not retrievable	Gives a value between 0 and 1, which illustrates a word’s spread across topics (0 means all occurrences are concentrated in one part of the corpus, 1 means occurrences are distributed evenly across all parts).

^a^The direction of the McDonald and Shillcock (2001) contextual distinctiveness metric differs from all others. To maintain consistency, we categorised high distinctiveness as low diversity and low distinctiveness as high diversity

### Behavioural studies

Most of the experiments included in this review investigated the direct influence of diversity on various lexical processing tasks and were classified as behavioural studies (111 out of 145 experiments).

#### Operationalisations and terminology of diversity

**Fig. 3:**
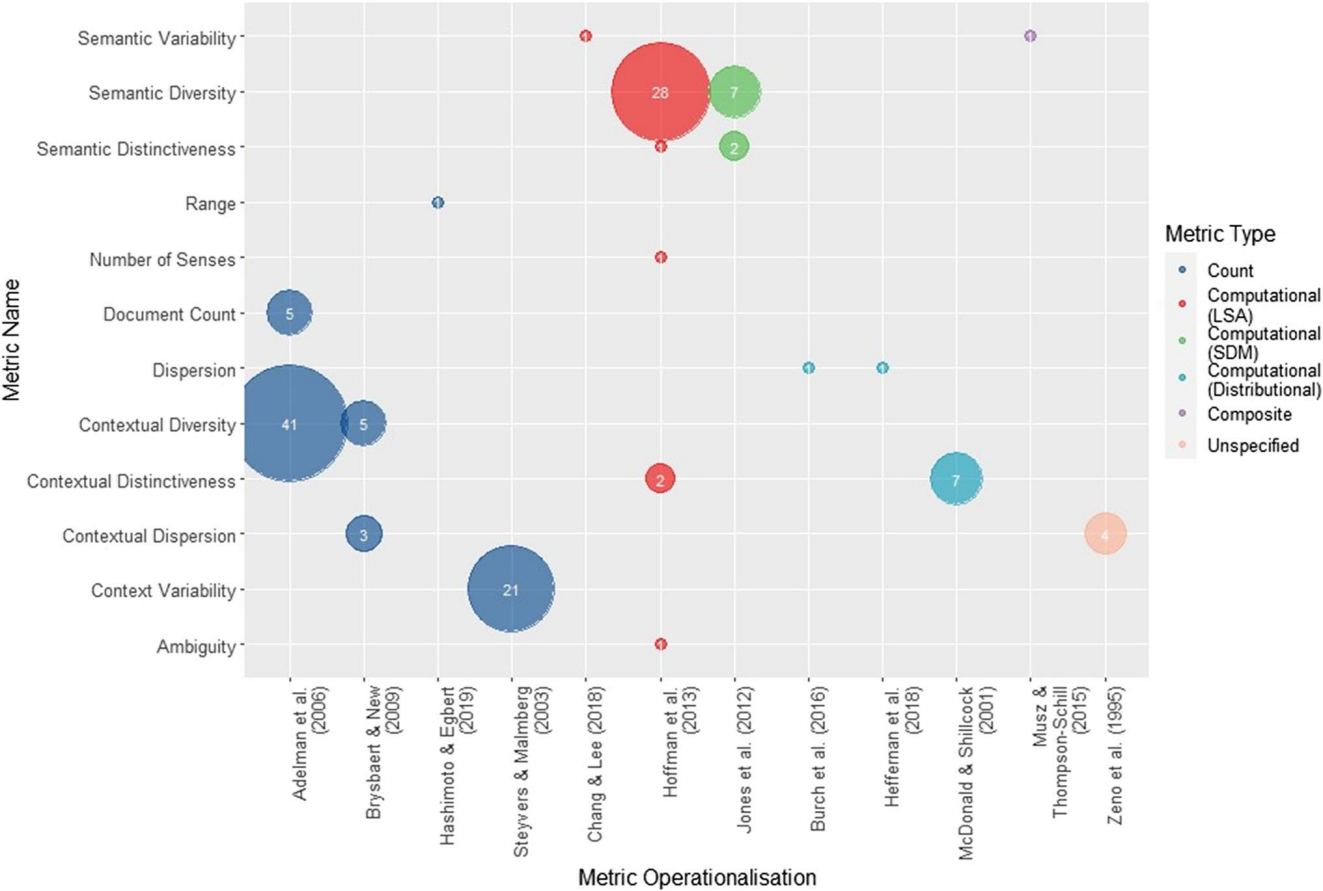
Behavioural studies—The number of times each operationalisation of diversity has been used and the different terms that have been used to refer to this broken down by metric type (Colour figure online)

Figure 3 shows the different names used across behavioural experiments for each diversity metric identified in this review (labelled according to the first author’s name from the study in which the metric was originally used). It shows the number of experiments that have adopted each term and metric, grouped by metric type. Where experiments used multiple diversity metrics, these have been counted separately (e.g., if both ‘contextual diversity’ and ‘semantic diversity’ were investigated in the same experiment, each metric has been treated separately within the count).

Diversity metrics were operationalised in 12 different ways (Table 2). There were also 12 different terms used to refer to diversity, and these did not always correspond to the same metric; some experiments adopted the same operationalisation of a metric but termed it something different^3^, indicating that terminology has been applied inconsistently. Of the 111 experiments included in this section, 57 varied diversity continuously, with 16 specifying the range of diversity values, and 54 varied diversity categorically, with 36 studies specifying values for the high- and low-diversity conditions.

#### Does diversity influence word-form processing, and what factors modulate this?

Fifty experiments contained at least one of the nine different tasks that were classified as a measure of word-form processing (Table 3 for further details).^4^ These tasks were grouped based on the type of knowledge and cognitive processes needed to perform them: I) familiarity judgement, II) word production, and III) other. Familiarity judgement tasks all require participants to indicate whether they know a word form or not (e.g., lexical decision). Word production tasks require participants to say a word out loud when prompted with its written or spoken form (e.g., word naming). Other tasks did not fall into either category (e.g., fragment completion).

**Table 3:** Descriptions of all tasks used to assess word-form processing and their task categories

Task type	Task	Description	Number of experiments
Familiarity judgement	Auditory lexical decision	Decide whether a sequence of phonemes is a word or not	2
	Lexical decision	Decide whether a string of letters is a word or not	42
	Primed lexical decision	Decide whether a string of letters following a prime is a word or not	1
	Signal-to-respond lexical decision	Decide whether a string of letters is a word or not when prompted by a salient signal.	1
	Vocabulary of American English Size Test	Yes/No vocabulary test: Decide whether string of letters is a word or not	1
Word production	Auditory word repetition	Repeat a spoken word out loud	2
	Word naming	Read a printed word out loud	13
Other	Auditory word recognition	Identify a spoken word presented in noise	1
	Speeded word fragment completion	Select one of two letters to complete a word missing one letter	1

**Fig. 4:**
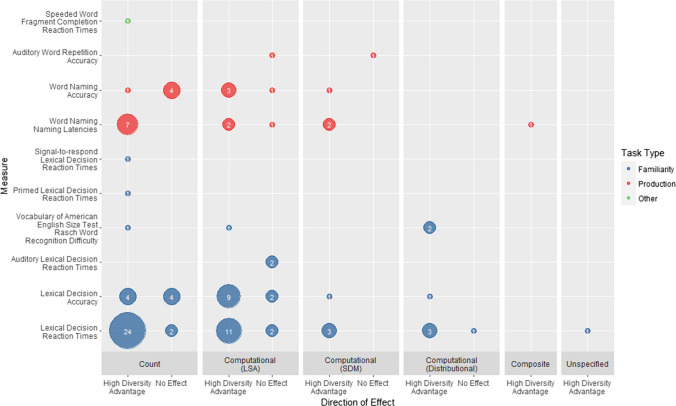
Number of reports of high-diversity advantage and no effect for each form processing measure broken down by metric type and task type. Within each task type tasks are ordered by frequency of usage

Figure 4 shows the pattern of diversity effects across metrics and task types. A high-diversity advantage is consistently reported across tasks and metrics (*N* = 81). No experiments found a low-diversity advantage, and no effect was only reported in a minority of instances (*N* = 20). The following section discusses the effects of diversity across metric operationalisations within each of the three task types described earlier. Where appropriate, we explore how methodological factors may have led to inconsistencies in the results, and what other experimental variables interacted with diversity.

##### Familiarity judgement

Familiarity judgements, which are all variations of the lexical decision task, were the most common type of task used to assess word-form processing (Table 3; 47 experiments). For the vocabulary test of American English size, the dependent measure was Rasch word recognition difficulty.^5^ For all other tasks the dependent measures were accuracy (14 experiments) and/or reaction times (46 experiments). Of the 47 experiments, 46 controlled for effects of word frequency in their stimuli or included it as an additional variable in the analysis. Other common control variables included concreteness, word length, and orthographic neighbourhood size (see data extraction form for full details). Due to the number of experiments included in this review and the range of control variables included, we only report effects of these variables when they significantly interact with diversity (RQ7) and do not report their main effects.

***Main effects of diversity*** In the standard visual lexical decision task, a high-diversity advantage on reaction times was reported 42 times, and no effect was reported five times (Fig. 4). There were two reports of no effect from experiments using count-based metrics (Hsiao & Nation, 2018, Experiment 1; Recchia & Jones, 2012, Analysis 3b). In Hsiao and Nation’s (2018) Experiment 1, lack of variation in diversity likely accounts for the null effect. The experimental stimuli were selected to systematically vary in LSA-derived diversity, and there was very little variation in count-based diversity, with low-diversity words (*N* = 30) having a mean value of 3.36 and high-diversity words (*N* = 30) having a mean value of 3.38. In Experiment 2, in which count-based diversity was explicitly varied over a larger sample of words (*N* = 300) and over a greater range of values (1.79–4.07), there was a significant high-diversity advantage. The null effect found in Recchia and Jones’s (2012) Analysis 3b may also be due to a lack of variation in the stimuli, which were concrete nouns taken from a large-scale feature norming study (McRae et al., 2005) that were not explicitly developed to vary in terms of diversity. In contrast, there was a high-diversity advantage in Analysis 1 in this paper, when stimuli were selected from the authors’ own norms and consisted of both concrete and abstract nouns chosen to have a range of diversity values.

No effect on reaction times was reported twice in experiments using an LSA-derived metric (Hsiao et al., 2020: Experiment 2; Sidhu et al., 2016, Experiment 1). Hsiao et al. (2020) found a high-diversity advantage for children, but not adults, on lexical decision times to 160 words taken from Hoffman and Woollams (2015). This is likely because these words varied much more in diversity for children (range: 1.28–3.65, calculated over the Oxford Children’s Corpus) than for adults (range: 1.42–2.16, calculated over the British National Corpus). The authors also acknowledge that the adult experiment was underpowered. Experiment 3 used a larger adult sample and the full 240-item set from Hoffman and Woollams and found the predicted high-diversity advantage. The null effect found by Sidhu et al. (2016) could be attributable to stimulus characteristics. The key feature of this experiment is that they used pure verb lists. Linzen et al. (2013) also used pure verb lists and found no effect of diversity, using a distributional metric (McDonald & Shillcock, 2001).

Turning to lexical decision accuracy, a high-diversity advantage was reported 14 times and no effect was reported six times.^6^ These null effects are likely attributable to ceiling effects due to high levels of accuracy on these tasks.

The vocabulary test of American English size (Hashimoto & Egbert, 2019) found a high-diversity advantage across operationalisations, including a count-based metric (Adelman et al., 2006), an LSA-derived metric (Hoffman et al., 2013), and two distributional computational metrics (Burch et al., 2016; McDonald & Shillcock, 2001).

The experiments using variations of the lexical decision task suggest that task demands can alter the effect of diversity on familiarity judgements. Two experiments reported no effect on auditory lexical decision times using LSA-derived diversity (Goh et al., 2016; Nenadić et al., 2019). This contrasts with the consistent high-diversity advantage for reaction times seen in experiments using visual lexical decision. However, Goh et al. (2016) selected their stimuli from McRae et al. (2005); thus, it may be that a lack of diversity variation within the stimuli underlies this null result. On the other hand, Nenadić et al. (2019) used 9,086 words from the Massive Auditory Lexical Decision (MALD) project (Tucker et al., 2019), which were much more varied in diversity. 7 It is possible that diversity effects do not emerge as auditory lexical decision is influenced to a greater extent by additional stimulus properties such as phonological uniqueness point. Alternatively, a word’s semantic history may affect auditory and visual processing differently.

The high-diversity advantage reported in the signal-to-respond (Hargreaves & Pexman, 2014) and primed (Adelman et al., 2014) lexical decision tasks only emerged under certain conditions. Both experiments operationalised diversity using count-based metrics. In signal-to-respond lexical decision, the effect of diversity increased with signal-to-respond duration and was only significant at 400 ms, whereas orthographic and phonological variables influenced responses at 100 ms and 200 ms but not at 400 ms. This suggests that only later processing is influenced by lexical/semantic variables such as count-based diversity. In primed lexical decision, high-diversity words showed larger priming effects but only when phonological Levenshtein distance was absent from the regression model.

***Interactions*** Only 10 familiarity judgement experiments reported significant interactions between diversity and other item-level (e.g., word frequency), or participant-level (e.g., age) variables. These studies indicate that the high-diversity advantage is greater for high- compared with low-frequency words, at least for LSA- or SDM-derived metrics (Chapman & Martin, 2021; Hsiao et al., 2020, Experiment 4; Jones et al., 2012), although Hamrick and Pandza (2020) found no effect using a count-based metric. In addition, individual studies indicate that that LSA-derived diversity effects may be magnified for words that are acquired relatively early (Hsiao & Nation, 2018, Experiment 2) or are low in imageability (Hsiao et al., 2020, Experiment 4). Evidence also suggests that count-based diversity effects may be reduced for longer words (Berger et al., 2019). Finally, Yap et al. (2015) report that LSA-derived diversity effects were larger when the stimuli were more degraded. This is consistent with the results of the speeded word fragment completion and auditory word recognition tasks (which also used degraded/partial stimuli), and suggests that participants need less information about a stimulus in order to make a familiarity judgement when that item is high diversity.

Turning to participant-level interactions, the focus has been primarily on how diversity effects are modulated by language experience. Hamrick and Pandza (2020) found that count-based and LSA-derived diversity had a similar influence on lexical decision reaction times regardless of whether participants were responding to items in their native language (monolinguals) or second language (highly proficient bilinguals). However, Johns et al. (2016) found that SDM-derived diversity accounted for significantly more variance in reaction times in bilingual compared to monolingual participants, and in older compared with younger adults.^8^ Johns et al. reported that SDM-derived diversity accounted for significantly more variance in English lexical decision reaction times for highly proficient English–French bilinguals than count-based diversity, echoing their findings for monolinguals (Jones et al., 2012). Finally, Skalicky et al. (2019) report that for count-based diversity, the high-diversity advantage for visual lexical decision times was weaker for participants with a greater (>8 years) compared with a lower (<1 year) length of English learning. They did not find a relationship between diversity as operationalised through a distributional computational metric (McDonald & Shillcock, 2001) and length of English learning, suggesting that beginning English learners benefit more from seeing a word in multiple different documents than from variations in the semantic content of those documents. Taken together, these studies provide a relatively complex picture as to how linguistic experience might modulate effects of diversity, with future work needed to replicate and integrate these findings.

##### Word production

Fourteen experiments assessed the effect of diversity on form processing using auditory word repetition or visual word naming (Fig. 4). All experiments controlled for word frequency in their stimuli or included it as an additional variable in their analysis.

***Main effects of diversity*** For visual word naming, three experiments reported both accuracy and latency, six reported only latencies, and three reported only accuracy. For naming latencies, a high-diversity advantage was reported 12 times, and no effect was reported once. The null result was reported in Sidhu et al. (2016) Experiment 3a using an LSA-derived metric. This study is distinct in that it is the only one to have used pure verb lists, whereas all others used either mixed lists or pure noun lists.

For naming accuracy, a high-diversity advantage was reported five times and no effect was reported five times. Three of the null results were from experiments within one study with children (Hsiao & Nation, 2018, Experiments 1 & 3), which employed one count-based metric (Adelman et al., 2006) and one LSA-derived metric (Hoffman et al., 2013) calculated over the Oxford Children’s corpus. In Experiment 1, performance was at ceiling and therefore not analysed further. Experiment 3 obtained an LSA-derived diversity advantage but there was no effect of the count-based measure. However, like in Experiment 1 and as discussed in relation to lexical decision, the item set was small and the authors report that there were very few words with low count-based diversity. The other null results were reported in Soares et al. (2019) and Yap et al. (2011). These were likely due to ceiling effects (mean accuracy >98% in both experiments) as the predicted high-diversity advantage was observed in naming latencies.

Word repetition was only used as an outcome measure in one experiment with semantic and nonsemantic aphasic patients (Chapman & Martin, 2021). No effect on accuracy was found for either LSA-derived or SDM-derived diversity. Given the absence of other experiments using this task, the reasons for this null finding are unclear.

***Interactions*** Only one study reported a significant interaction between diversity and another variable. Consistent with the earlier effects on familiarity judgements, Chapman and Martin (2021) found that the high-diversity advantage was greater for high- than low-frequency words. For LSA-derived diversity this was significant for word naming accuracy but not latencies. For SDM-derived diversity the interaction was significant for naming latencies but not accuracy. The authors suggest that these differing patterns of results reflect the different correlations between the two metrics and their frequency measure.

##### Other form-based outcome measures

Two experiments (Fig. 4) utilised tasks that involved identifying familiar but degraded word forms that did not fall into either the familiarity judgement or word production categories: auditory word recognition (Johns et al., 2012) and speeded word fragment completion (Heyman et al., 2016). Accuracy and/or reaction times were the dependent measures in both tasks. Only Johns et al. (2012) controlled for word frequency.

For auditory word recognition, Johns et al. (2012) reported that both count-based and SDM-derived diversity account for significant unique variance in accuracy. The direction of the diversity effect was not reported. For speeded word fragment completion (Heyman et al., 2016), a significant high-diversity advantage for reaction times using a count-based metric was found. However, it is unclear whether this result is attributable to diversity or word frequency, which was not controlled.

No significant interactions with other variables were reported. Future studies are clearly needed to assess whether the consistent main effects of diversity, and patterns of interactions, can be seen with these alternative methodologies that are currently underused by the field.

##### Conclusions

The 46 experimental studies included in this review, in which stimuli are presented *visually*, show a highly consistent, facilitatory effect of diversity across word-form processing tasks*.* Most null effects are attributable to a lack of diversity variation within the stimuli or near-ceiling levels of accuracy. Importantly, effects are extremely robust across different tasks and different operationalisations of diversity.

In contrast, there is more limited evidence that diversity effects generalise to *auditory* processing. Only four experiments investigated word-form processing using auditory stimuli, and the only significant effect of diversity comes from a study in which participants identified words in noise (Johns et al., 2012). It is unclear whether these null results reflect theoretically important modality differences or are the consequence of either task limitations or the use of diversity measures derived from *written* corpora, which may not accurately reflect the characteristics of spoken language. This review emphasises the need for further research using auditory stimuli with more sensitive measures of both diversity and performance.

In contrast to the compelling evidence concerning the main effects of diversity, data concerning how these effects are modulated by other factors is sparse. Most of the experiments included in this review controlled for the effects of word frequency or included it as an additional variable in their analyses. Diversity effects are consistently found over and above those of word frequency, suggesting that diversity has an independent influence on word-form processing. There is also relatively consistent evidence across both familiarity and production tasks that, when diversity is operationalised with measures that take semantic information into account (e.g., Chapman & Martin, 2021; Jones et al., 2012), the diversity effects are greater for high- compared to low-frequency words. These findings support the view that when a repetition of a word is accompanied by a change in semantic context, this leads to greater updating of its strength in memory, compared with if it had been seen in the same context, which then confers an advantage on form processing tasks. While this review has found preliminary evidence that other item-level characteristics (e.g., age-of-acquisition, imageability, length, stimulus degradation) may also interact with diversity, results come from single experiments and need replicating in studies that fully account for a wider range of potential predictors before firm theoretical conclusions can be drawn.

The reported interactions between diversity and participant-level characteristics indicate that although diversity may be a general organisational principle of the lexicon, linguistic experience may modulate its importance. This is evident in studies comparing bilingual participants to monolingual participants (Hamrick & Pandza, 2020; Johns et al., 2016), as well as those that explore effects of age and proficiency (Hsiao & Nation, 2018; Skalicky et al., 2019). However, as with the item-level effects, these conclusions rely on findings from single studies, and require replication.

#### Does diversity influence word-meaning processing, and what factors might modulate this?

Thirteen experiments contained at least one of the 11 different tasks that were classified as a measure of word-meaning processing (see Table 4)^9^. We again grouped tasks into three categories, based on the type of knowledge and cognitive processes needed to perform them: semantic decision, picture naming, and qualitative semantic tasks. Semantic decision tasks involved speeded judgements about the semantic properties of the words (e.g., concreteness decision). Picture-naming tasks involved producing a single word to describe a picture. In qualitative semantic tasks, participants provide ratings or relatively unconstrained responses (e.g., word association), which provided qualitative insights into their interpretations of the words rather than measuring how quickly or accurately word meanings are processed. Unless otherwise specified, all tasks were administered visually. All experiments controlled for the effects of word frequency in their stimuli or included it as an additional variable in their analysis. Full details of other variables included or controlled for in the analyses are available in the data extraction form. Again, we only discuss these additional variables where they show a significant interaction with diversity and do not report their main effects.

**Table 4:** Descriptions of all tasks used to assess word-meaning processing and their task categories

Task type	Task	Description	Number of experiments
Semantic decision	Auditory concreteness decision	Decide whether a word presented aurally is concrete or abstract	1
	Concreteness decision	Decide whether a word is concrete or abstract	3
	Cross-modal definition matching	Judge if an auditory definition matches visually presented target word in meaning	1
	Signal-to-respond concreteness decision	Make a concrete/abstract decision to a word in response to a salient signal	1
	Semantic relatedness decision	Judge whether two words are related	3
Picture naming	Object naming	Name an object seen in a picture	1
	Picture naming	Name the object or action seen in a picture	1
Qualitative	Context availability ratings	Rate on 7-point scale how difficult it is to think of a context (imagined situation or circumstance) associated with a word	1
	Creative word generation	Generate creative words (words that are linked to the target, but that very few, if any, other people would come up with) in response to a cue word	3
	Sentence availability ratings	Rate on 7-point scale how difficult it was to think of a sentence for a given word	1
	Word association	Generate up to 3 single-word responses to a cue word	1

**Fig. 5:**
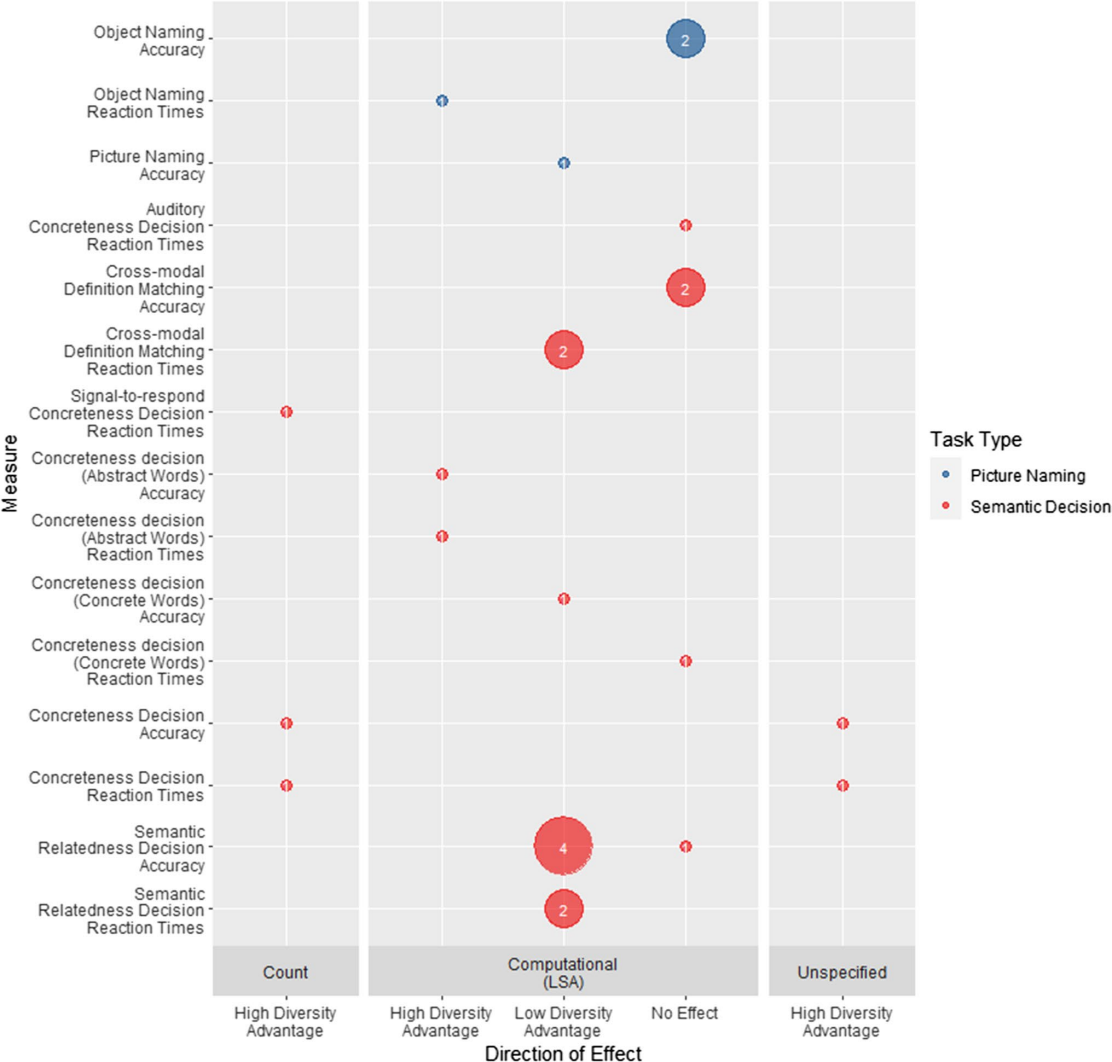
Number of reports of high-diversity advantage, low-diversity advantage, and no effect for each meaning-processing measure broken down by metric type and task type. Within each task type tasks are ordered by frequency of usage. (Colour figure online)

Figure 5 shows the pattern of results across task across metrics and task types.^10^ The results of experiments investigating meaning processing are less consistent than those investigating form processing. Most experiments have used LSA-derived metrics with some reporting a high-diversity advantage (*N* = 3), some a low-diversity advantage (*N* = 10), and some no effect (*N* = 7). Experiments that operationalised diversity using count-based (*N* = 3) or unspecified (*N* = 2) measures found a consistent high-diversity advantage. We next consider how changing task demands, metric operationalisations, and additional experimental variables influence diversity effects within the different task type categories. Where relevant, we will also report on quality issues that may have impacted the results.

##### Semantic decision

Speeded semantic decision, particularly concreteness decision, was the most common task type used to examine the effect of diversity on word-meaning processing (Table 4; nine experiments).

***Main effects of diversity*** A high-diversity advantage was found in the two studies that used a standard visual concreteness decision task together with a count-based diversity metric (Yap et al., 2011) or an unspecified diversity metric (Pexman et al., 2008), suggesting that words occurring in a greater number of documents may enjoy a word-meaning processing advantage. Hargreaves and Pexman (2014, Experiment 2) also found a count-based diversity advantage in signal-to-respond concreteness decision, though this increased across signal-to-respond durations and was only significant at 400 ms. However, semantic variables (number of senses, metrics relating to number of neighbours) had an early facilitatory effect on processing at 100 ms and 200 ms. This suggests that count-based diversity has a relatively late influence on concrete/abstract decisions but other semantic properties of words have an earlier influence.

In contrast, results on concreteness decisions are more mixed for metrics that account for the semantic content of the contexts in which words appear, with diversity having different effects for the concrete and abstract words. Using LSA-derived diversity, Chapman and Martin (2021) found a null effect for reaction times, but a low-diversity advantage for accuracy, for decisions to concrete words. For abstract words, they obtained a high-diversity advantage in both accuracy and reaction times. For auditory concreteness decision (Goh et al., 2016), there was no effect of LSA-derived diversity for concrete words, responses to the abstract words were not analysed. Future studies are needed to determine whether this complex pattern of results replicates, before considering whether they can be explained by current theories.

In contrast, the three studies that used semantic relatedness judgements together with an LSA-derived diversity metric reported a consistent low-diversity advantage in healthy controls (Hoffman et al., 2011, 2013; Hoffman & Woollams, 2015, Experiment 2). Hoffman et al. (2013) also found a low-diversity advantage for accuracy in semantic aphasia patients, whereas Hoffman et al. (2011) found no effect of diversity for semantic dementia patients. A similar low-diversity advantage for LSA-derived diversity is seen for cross-modal definition matching reaction times for both adults and children, though accuracy was at ceiling (Hsiao et al., 2020, Experiment 1). Taken together, these results suggests that, at least for healthy controls, making precise meaning judgments is more difficult for words that are experienced in more varied semantic contexts.

***Interactions*** Only two experiments reported significant interactions between diversity and other variables on semantic decisions. Hsiao et al., (2020, Experiment 1) found that high LSA-derived diversity was associated with a greater increase in reaction times for high- than low-frequency words, such that the advantage for high-frequency words in definition matching was eliminated as diversity increased. The influence of relatedness—that is, whether the two words or word and definition for which participants made a decision were similar in meaning or not—was investigated in two experiments using LSA-derived diversity. For semantic relatedness decision, Hoffman and Woollams (2015) found that adults made significantly more errors for high- than low-diversity words on related trials, but not on unrelated trials. However, for cross-modal definition matching, Hsiao et al. (Experiment 1, adults) found a low-diversity advantage for response times on unrelated trials, but not on related trials. The source of this discrepancy is unclear and needs further investigation.

##### Picture naming

***Main effects of diversity*** There is no consistent pattern of diversity effects on object-naming/picture-naming tasks. For control participants, Chapman and Martin (2021) found a high LSA-derived diversity advantage on object-naming reaction times but not accuracy, but there was no significant effect of diversity on either measure for aphasic patients. In contrast, using the same metric, Alyahya et al. (2020) found a low-diversity advantage for picture-naming accuracy in their aphasic sample. No significant interactions between diversity and other variables were reported.

##### Qualitative semantic tasks

Finally, we discuss tasks that do not provide a directional diversity effect but do give qualitative insights into participants’ interpretation of word meaning; word association, creative word generation, and context and sentence availability ratings.

Van Rensbergen et al. (2015) explored whether word association responses given to low and high count-based diversity cues corresponded in terms of diversity. There was no effect of cue diversity on response diversity. Three experiments used a word-association task in which participants were explicitly instructed to generate creative words^11^ in response to high and low LSA-derived diversity cues (Johnson & Hass, 2022). Experiment 1 indicated that high-diversity cue words lead participants to produce responses that were more varied in their diversity values and from more diverse contexts.^12^ Experiment 2 included an additional measure to explore whether participants’ pattern of responding indicated that they were exploring distinct semantic categories.^13^ They found that although high-diversity cues lead to more varied responses, there was no indication that this led to responses from more diverse contexts or more distinct semantic categories. However, when replicated in Experiment 3 with better controlled stimuli, they found high-diversity cues led to participants generating responses from more diverse contexts and exploring more distinct semantic categories. Furthermore, in both Experiments 1 and 3, the variability of responses was mediated by the extent to which participants generated responses from more diverse contexts. This suggests that diversity of cue words, as captured through LSA-derived measures, influences the variability of response words by encouraging participants to explore more diverse contexts. However, count-based diversity does not have a similar effect.

Context and sentence availability ratings (Taylor et al., 2022) are obtained by presenting participants with words and asking them to rate how easy it was to think of a situation (context availability) or sentence (sentence availability) in which the word could be used. Words higher in count-based diversity resulted in higher context and sentence availability ratings than words lower in count-based diversity. On the other hand, LSA-derived diversity was not significantly related to context availability but was positively correlated with sentence availability. This supports the idea that words high in count-based diversity are generally more accessible (Adelman et al., 2006) but suggests that although words high in LSA-derived diversity are easy to place in sentences, this does not influence how easy it is to think of a situational context in which that word is used.

In summary, very few studies have adopted qualitative tasks. Future studies are needed to replicate these findings and to establish how the results of these tasks relate to performance on quantitative tasks of word-meaning processing, and how they tie in with theoretical predictions.

##### Conclusions

In contrast to the consistent high-diversity advantage seen for word-form processing, the effects of diversity on word-meaning processing seem to depend on both operationalisation and task. Where diversity has been operationalised through count-based metrics, we see a high-diversity advantage like that seen for word-form processing. However, only concreteness decision performance has been investigated with these metrics, making it difficult to draw firm conclusions about the generalizability of these effects. Most research has operationalised diversity using LSA-derived metrics, which account for the semantic contexts in which words occur, and here the results are more mixed. Concreteness decision and picture naming experiments show no clear pattern of effects. In contrast, semantic relatedness decision and definition matching tasks show a consistent low-diversity advantage. All these effects are observed over and above effects of word frequency, but further work is needed to establish how different diversity metrics influence performance on different semantic tasks and to establish how these interact with other variables, such as concreteness, imageability, and polysemy.

#### Does diversity influence other behavioural outcome measures, and what factors might modulate this?

Fifty-five experiments using 18 different tasks contained at least one outcome measure that could not be straightforwardly classified as a measure of either word-form or word-meaning processing (Table 5). In some cases, these related to other aspect of cognitive processing (e.g., memory), while other outcome measures were likely influenced by *both* form and meaning processing (e.g., eye-tracking). Grouping tasks based on the knowledge and/or cognitive processes needed to perform them resulted in three main categories: recognition memory, recollective memory, and eye-tracking. Recognition memory tasks involved making a speeded judgement regarding whether a word had been encountered during training. Recollective memory tasks involved recalling specific properties of words seen during training (e.g., recalling order of presentation). Eye-tracking studies involved monitoring participants’ eye movements during reading. All other tasks that did not fall into one of these categories are reported separately in the supplementary materials (https://osf.io/9h7ac), since most were only used once and they currently do not make significant theoretical contributions. Unless otherwise specified, all tasks were administered visually.

**Table 5:** Descriptions of all other outcome measures and their task categories

Task type	Task	Description	Number of experiments
Recognition memory	Associative recognition	Decide whether two words were presented together in training	3
	Auditory recognition memory	Decide whether word presented aurally was presented in training	1
	Mouse tracking	Indicate whether word was seen in training by moving a mouse	1
	Recognition memory	Decide whether word presented visually was presented in training	14
Recollective memory	Cued recall	Recall the word a cue word was presented with in a study phase	4
	Delayed free recall	Recall as many words from a studied list as possible after delay	1
	Free recall	Recall as many words from a studied list as possible	7
	Serial list recall	Recall as many words from a studied list as possible in the order of presentation	8
Eye-tracking	Eye-tracking	Eye movements monitored during sentence reading	8
Other	Autobiographical memory interview	Describe life events that were striking or memorable	1
	Corpus analysis	Analysis of case-marker omission rate as a proxy for processing effort in a corpus of natural language	1
	EEG	Recordings taken from electrodes placed across the scalp	2
	fMRI	Brain scans taken using an MRI scanner	1
	Past tense generation	Pronounce the past tense form of a verb	1
	MEG	Neural activity measured via magnetoencephalography	1
	Semistructured interviews	Interviews conducted with specific elicitation materials to prompt conversation	1
	Syntactic classification	Decide whether word presented is a noun or a verb	1
	Test-based age of acquisition	Determine whether children of different ages can match word to 1 of 3 meanings	1

**Fig. 6:**
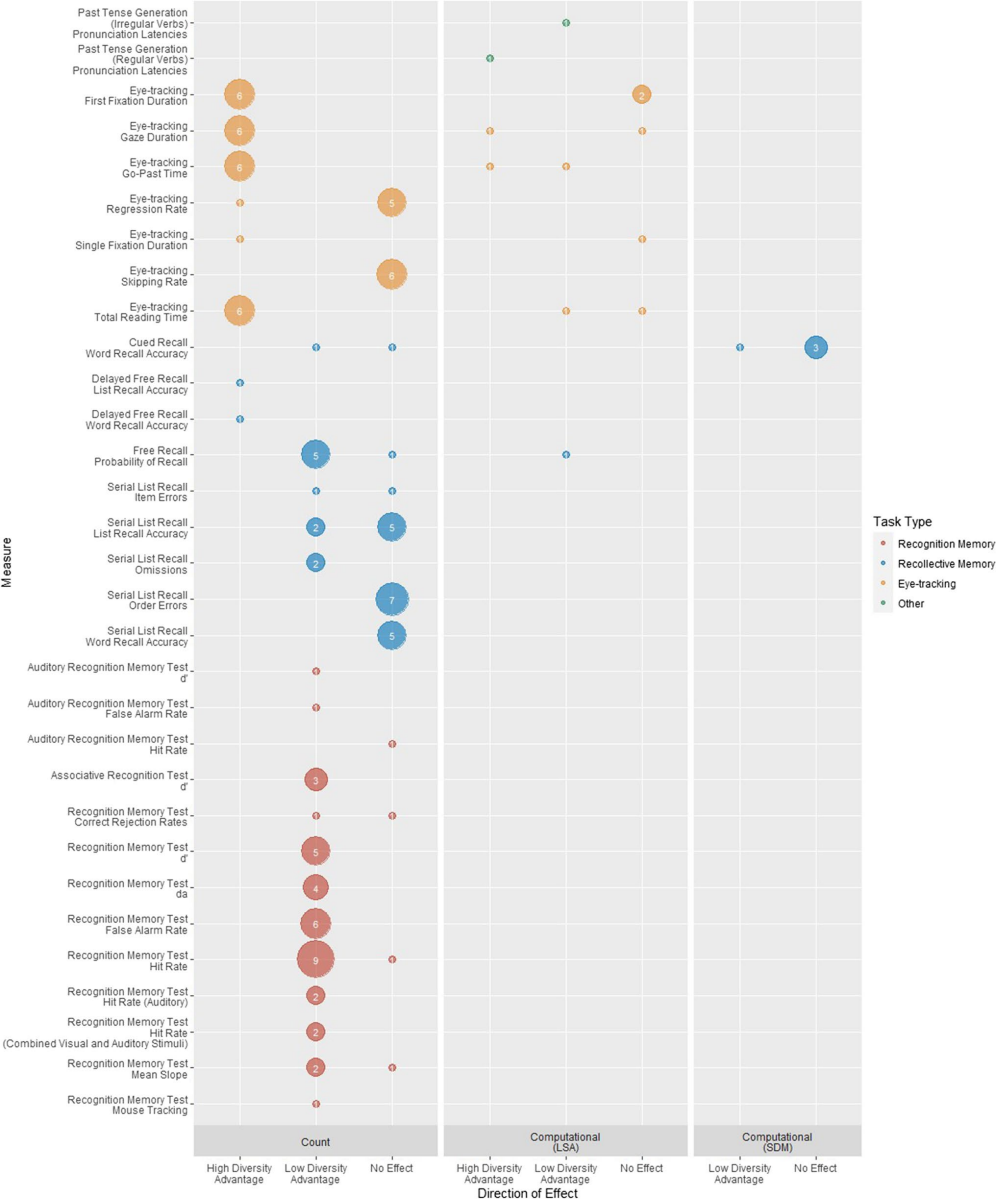
Number of reports of high-diversity advantage, low-diversity advantage and no effect for each other outcome measure used in the behavioural studies broken down by metric type and task type. Within each task type tasks are ordered by frequency of usage. (Colour figure online)

Figure 6 shows the pattern of diversity effects across tasks and metric types.^14^ Most experiments operationalised diversity through count-based metrics, with LSA- and SDM-derived computational metrics used in a minority of instances.

##### Recognition memory

Three tasks were used across 15 experiments to assess recognition memory: recognition memory test, auditory recognition memory test, and associative recognition test (Table 5). Participants studied words that varied in diversity and then indicated in a test phrase whether they had seen them or not. Only count based metrics have been used to assess the effect of diversity on recognition memory (Fig. 6) using a range of dependent measures.^15^ All studies reported controlling for word frequency in their stimuli or included it as an additional variable in their analysis.

***Main effects of diversity*** Figure 6 shows that there is a consistent low-diversity advantage for recognition memory across all dependent measures, with no effect reported in just four instances. No studies reported a high-diversity advantage. This indicates that low-diversity words are more memorable than high-diversity words. The low-diversity advantage has mostly been seen for tasks in which participants judge the familiarity of isolated words, but Aue et al., (2018, Experiments 1 & 2) report a similar effect when the familiarity of word pairs is judged. Insight into this pervasive low-diversity advantage comes from Cook et al. (2006). When making familiarity decisions, participants were also asked to judge whether they ‘remembered’ an item (conscious recollection) or ‘knew’ it (general familiarity). They found that the proportion of ‘remember’ responses was significantly higher for low versus high-diversity items, whereas the proportion of ‘know’ responses was significantly higher for high versus low-diversity items (see also Meeks et al., 2014, Experiments 2 and 3). These results suggest that the low-diversity advantage in recognition memory tasks may be related to the memorability or distinctiveness of the items, rather than general familiarity. Consistent with this view, two recognition memory experiments suggest that low diversity also improves source memory (e.g., identifying the item’s presentation modality; Cook et al., 2006; Marsh et al., 2006a). The authors suggest that low-diversity words have fewer preexisting associations, allowing participants to strongly associate them with characteristics of the encoding experience. Cook et al. (2006) found that when stimuli were presented visually at training and aurally at test, the low-diversity advantage was attenuated, suggesting that the modality switch attenuates participants’ ability to recall specific properties of the studied items, which reduces the low-diversity advantage.

However, it is important to note that all 14 studies reporting a low-diversity advantage on recognition memory used the same stimuli, taken from Steyvers and Malmberg (2003). These high- and low-diversity stimuli differed on several dimensions, including number of orthographic neighbours, concreteness, and age of acquisition, and, although they were equated on TASA frequency, items differed on SUBTLEXus and CELEX frequencies, with low-diversity words also having lower frequencies than high-diversity words (Neath et al., 2022). It is well established that low-frequency words enjoy an advantage on recognition memory tasks (Glanzer & Bowles, 1976), so it is possible that the low-diversity advantage is instead attributable to word frequency. Indeed, Neath et al. (2022, Experiment 1) found no effect of diversity on recognition memory using words from Guitard et al. (2019) that were matched on multiple frequency measures including SUBTLEXus and CELEX. However, when they repeated their experiment using the Steyvers and Malmberg stimuli, they replicated the low-diversity advantage.

Two further experiments found no effect of diversity on recognition memory. Despite using the same stimuli as Steyvers and Malmberg (2003), Meeks et al., (2014, Experiment 2) reported a null effect on correct rejection rates, and Neath et al. (2022, Experiment 2) found no effect of diversity on mean *Z-*ROC slopes. It is unclear why these results contrast with others using this stimulus set.

***Interactions*** Cook et al. (2006) and Steyvers and Malmberg (2003) report that the high-diversity disadvantage was significantly greater for high-frequency words, whereas the low-diversity advantage was significantly greater for low-frequency words. They suggest that items that were both high diversity and high frequency were harder to remember because they have particularly low memorability or distinctiveness.

Several studies examined interactions between diversity, word frequency, and task-specific factors (encoding and test condition, Aue et al., 2018; study time, Cook et al., 2006; visual vs aural presentation, Marsh et al., 2006a; lure type, Papesh et al., 2019). These suggest that diversity effects differ according to test type, presentation modality, and study time, and can be affected by the distribution from which targets and lures are drawn.^16^ However, as all these studies used the stimuli of Steyvers and Malmberg (2003), it is not clear whether their results are driven by diversity or a combination of the other factors not controlled for in these stimuli.

***Conclusions*** In summary, the results suggest that low diversity provides a general advantage on recognition memory tasks. This may be because high-diversity words have several nonspecific memories associated with them, resulting in multiple weakly activated memory traces when they are encountered in training and at test (Aue et al., 2018; Meeks et al., 2014; Steyvers & Malmberg, 2003). A closely related explanation is that, by virtue of appearing in fewer contexts, low-diversity words have fewer preexisting associations, making it easier to form new associations with other experimental items or contexts, leading to a memorability advantage (Cook et al., 2006; Marsh et al., 2006a). However, because many of these experiments used the stimuli of Steyvers and Malmberg (2003), these conclusions should be taken with caution until the results are replicated with different, well-matched stimuli (see Neath et al., 2022). Indeed, emerging evidence presented in the computational modelling section using megastudy datasets and alternative diversity metrics (SDM-derived diversity) indicates that high diversity may instead provide an advantage for recognition memory (Johns & Jones, 2022).

##### Recollective memory

Four tasks were used to assess the effects of diversity on recollective memory: cued recall, delayed free recall, free recall, and serial list recall (Table 5). Similar to recognition memory, most experiments employed count-based operationalisations of diversity, with SDM- and LSA-derived computational metrics used in a minority of instances (Fig. 6). Nineteen of the 20 experiments controlled for word frequency in their stimuli or included it as an additional variable in their analysis.^17^

***Main effects of diversity*** The four experiments that used cued recall as an outcome measure produced one report of a low-diversity advantage and four reports of no effect, across various diversity metrics. For count-based diversity, Criss et al. (2011) found no effect in Experiment 3 but, in Experiment 4 they found that low-diversity cue words resulted in more accurate recall of targets than high-diversity cues. Studies using SDM-derived diversity found no effect of cue diversity on recall performance (Mak & Twitchell, 2020; Qiu & Johns, 2020).

In the seven experiments with free recall as an outcome measure, a similar low-diversity advantage was reported five times for count-based diversity and once for LSA-derived diversity (Goette et al., 2022). In contrast, no effect for count-based diversity was reported in an experiment in which the encoding task required participants to generate a similarity (e.g., shared feature) between the current and previously presented word (Marsh et al., 2006b, Experiment 3). This suggests that explicit formation of associations between words can attenuate the low-diversity advantage. One experiment used delayed free recall and reported a high-diversity advantage for count-based diversity (Aka et al., 2021) for recall of individual words and of the lists the words had appeared in. However, all six of the experiments investigating the effect of count-based diversity on (nondelayed) free recall used the Steyvers and Malmberg (2003) stimuli, whereas Aka et al. (2021) created their own word lists. It may be that, as with recognition memory, the consistent low-diversity advantage is an artefact of other properties of these stimuli.

Serial list recall was used as an outcome measure in seven experiments that all used count-based diversity. Parmentier et al. (2017) found that participants recalled more words in the correct position and made fewer omissions for low- than high-diversity lists (Experiments 1 & 2). They found no effect of diversity on order errors in either experiment and significantly fewer item errors for low-diversity lists in Experiment 1, but no effect of diversity on item errors in Experiment 2.^18^ However, when Guitard et al. (2019) attempted to replicate these findings in a series of five experiments using stimuli that were equated on more dimensions, they found no effect of diversity on list recall accuracy, word recall accuracy, or order errors, suggesting that a combination of uncontrolled variables drove Parmentier et al.’s results.

***Interactions*** Five experiments reported significant interactions with item-level variables (word frequency), task-specific factors, or participant-level variables (age).

In Experiment 3, Criss et al. (2011) found that the high-frequency advantage was greater for low- than high-diversity word pairs. In Experiment 4, the benefit of low-diversity cue words was greater for high- compared with low-frequency pairs. Thus, recall may be best for words that are both low diversity and high frequency.

Two experiments reported an interaction between diversity and task-specific factors (Marsh et al., 2006b, Experiments 1 & 2). In both experiments, when study and test occurred in the same context (same room or both in silence), there was a recall advantage for low-diversity items, but, when the environmental context was altered between study and test (different rooms or study in silence but test with music), recall did not differ for high- and low-diversity items. Marsh et al., (2006b) suggested that low-diversity items, as a virtue of appearing in fewer preexperimental contexts, form stronger item-to-environment associations during encoding, which are then used to cue recall. Changing the environment disrupts these associations and eliminates the low-diversity advantage.

Qiu and Johns (2020) found a low SDM-derived diversity advantage on recall accuracy for older adults (45–50 years old) but no diversity effect for younger adults (18–29 years old). The authors suggested that older relative to younger adults have accumulated more preexisting associations about high-diversity words and therefore find it difficult to form new arbitrary associations for them. It should be noted that when Mak and Twitchell (2020) replicated this experiment they also failed to find a diversity effect when they replicated this experiment with a younger adult sample (mean age 24.4 years).

***Conclusions*** In summary, there is weak evidence for a low-diversity advantage across cued recall, free recall, and serial list recall. Some authors have suggested that recall may be improved for low-diversity words due to them appearing in fewer and/or more specific contexts and having fewer preexisting associations to other words, which may make it easier to form new arbitrary associations to other words (e.g., Criss et al., 2011). However, the evidence presented here suggests that, like recognition memory, these effects may be limited to particular sets of stimuli that were not matched on key variables (see Aka et al., 2021; Guitard et al., 2019).

##### Eye-tracking

**Fig. 7:**
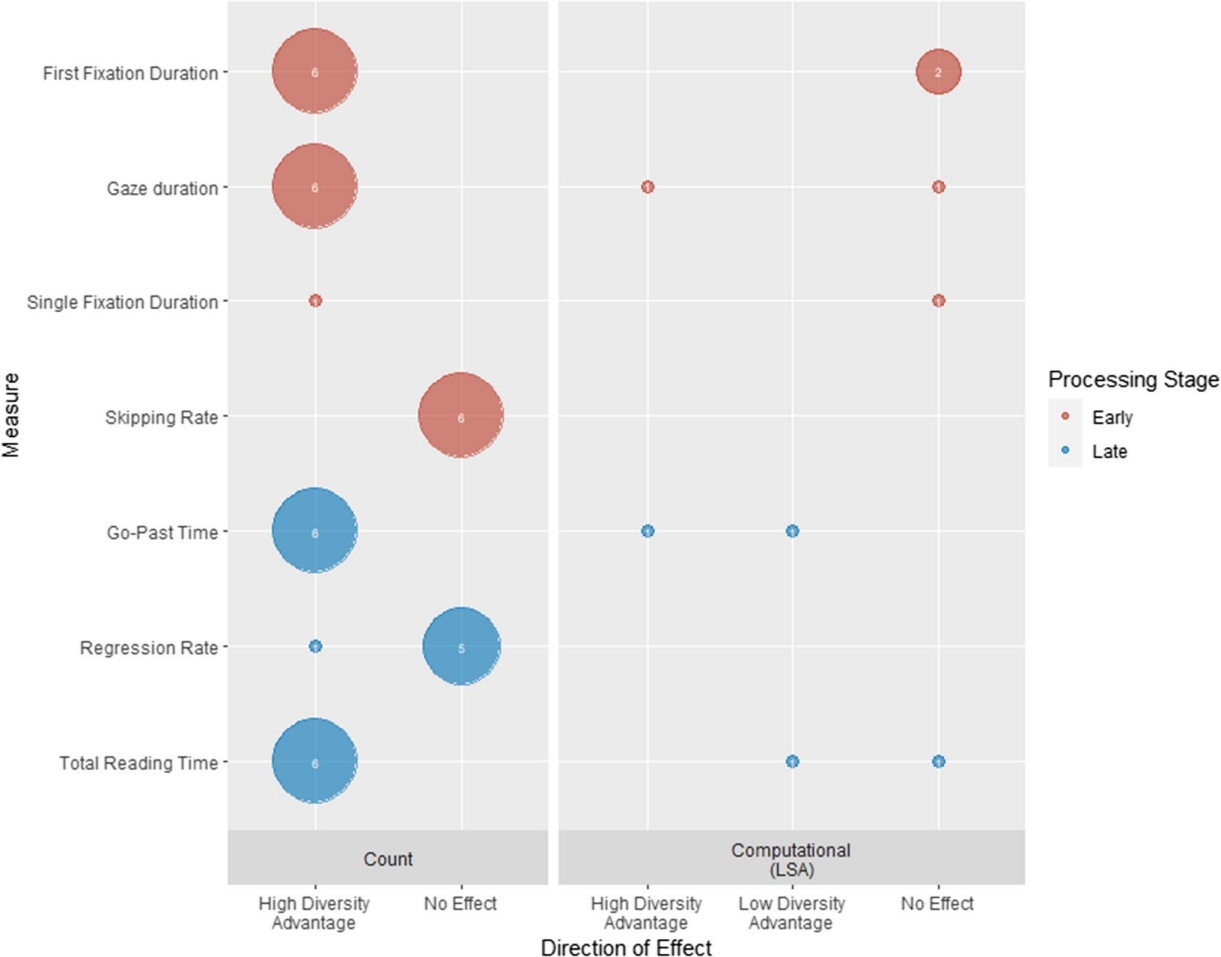
Number of reports of high-diversity advantage, low-diversity advantage, and no effect for each eye-tracking measure broken down by metric type and processing stage. (Colour figure online)

Dependent measures from eye-tracking studies were used in seven experiments. These can be broadly divided into early- and late-stage measures (Fig. 7). Early-stage measures are thought to reflect processes such as word identification, whereas late-stage measures are thought to reflect processes such as integrating words with the wider text (Clifton et al., 2007). Most studies investigating the effects of diversity on eye-movements have employed count-based measures, with LSA-derived metrics used in a minority of cases (Fig. 7). All experiments either controlled for word frequency in their stimuli or included it as an additional variable in their analysis, suggesting that these patterns of results can be reliably attributed to diversity effects.

***Main effects of diversity*** Experiments using count-based metrics (Fig. 7) show that high-diversity words received shorter first-fixation durations and gaze durations (early processing), and shorter go-past times and total reading times (later processing) than low-diversity words. This indicates that they are both easier to identify and integrate with the wider text than low-diversity words. Furthermore, these effects were robust for both English (Plummer et al., 2014) and Chinese stimuli (Chen et al., 2017a, 2017b).

The results for the two experiments using LSA-derived metrics are less consistent. Pagán et al. (2020) found a high-diversity advantage for gaze duration and go-past time, but no diversity effect on total reading time. Appearing in more semantic contexts may therefore facilitate initial word identification and integration into new contexts. On the other hand, Plummer et al. (2014) found a low-diversity advantage on total reading time and go-past time, but no effect of diversity on single fixation duration, first fixation duration, or gaze duration. However, the results of Plummer et al. should be interpreted with caution as stimuli were not chosen to explicitly vary in LSA-derived diversity, unlike those of Pagán et al. (2020).

No significant interactions between diversity and other variables were reported.

***Conclusions*** These results suggest that appearing in more contexts benefits word processing in sentences. There is converging evidence that high count-based diversity confers an advantage for both early- and late-stage eye-movement measures and some evidence to suggest that this is also the case for LSA-derived diversity.

### Computational modelling studies

Twenty experiments directly compared different diversity metrics in terms of how much variance they explain in behavioural data. Diversity metrics (Table 2) were computed over different materials, or the same materials organised differently, and were then applied to behavioural data derived from existing datasets. Details of the materials and datasets used can be found in the full database of included experiments available on the OSF (https://osf.io/ys6ne).

#### Operations and terminology of diversity

A total of 20 different diversity metrics were used (see Table 2), and the performance of these diversity metrics has been examined on several different behavioural outcome measures. We grouped these outcome measures using the same classification as in the behavioural studies section: I) word-form processing (RQ 4), II) other behavioural outcomes (RQ 9). No modelling studies investigated the effect of diversity on word-meaning processing. Within each category, the experiments focus on how much variance in the behavioural data is explained by the different diversity metrics. In doing so, they move beyond the simple question of whether diversity influences lexical processing (addressed in the behavioural studies section) and provide a more nuanced assessment of how and why diversity might impact lexical organisation. For ease of comparison, we have organised the sections addressing each research question by metric type. All models accounted for the effects of word frequency.

#### Word form processing

Sixteen experiments explored how much variance in word-form processing different diversity metrics explain. Eight behavioural measures of form processing were used; lexical decision reaction times and accuracy, word naming reaction times and accuracy, word prevalence ratings and reaction times, word familiarity ratings, and idiom familiarity ratings.^19^ A further five experiments specifically examined whether previously reported ambiguity effects on lexical decision could be explained by diversity (Cevoli et al., 2021; Hoffman et al., 2021).

Only nine of these 21 experiments reported a directional effect of diversity, with a consistent high-diversity advantage seen across all diversity metrics for lexical decision and word-naming latencies (Cevoli et al., 2021; Hoffman et al., 2021; Hollis, 2017; Johns & Jones, 2022; Johns et al., 2020; Johns, 2021b; Jones et al., 2012), word prevalence ratings and decision times (Johns & Jones, 2022; Johns et al., 2020; Johns, 2021b), and word and idiom familiarity ratings (Senaldi et al., 2022). These findings suggest that, consistent with the results reported in the Behavioural Studies section, words and phrases higher in diversity are easier to access, known by more people, and are perceived to be more familiar than low-diversity words and phrases.

We now consider how these experiments provide evidence for significant effects of diversity over and above effects of word frequency (as seen for the behavioural studies that investigated word-form processing) as well as informative comparisons between different diversity metrics.

##### Count-based metrics

In the earliest study of diversity effects on word-form processing, Adelman et al. (2006) showed that count-based diversity accounted for more variance in lexical decision and word naming times than word frequency. In a follow-up study, Adelman and Brown (2008) further demonstrated the superiority of count-based diversity over word frequency using eight transformations of their original metric^20^ using data from four corpora.^21^ These, in addition to untransformed count-based diversity, were then regressed against four lexical decision reaction time datasets (Balota et al., 1999, young adults; Balota et al., 1999, older adults; Murray & Forster, 2004, Experiment 1, English Lexicon Project). In 117/144 of these analyses, contextual diversity explained more variance than word frequency, supporting Adelman et al.’s (2006) original conclusion that contextual diversity not word frequency underlies variance in lexical decision times. Adelman and Brown suggested that this is due to the principle of ‘likely need’: that is, if a word appears in more varied contexts the more likely it is to be needed in a new context. This ‘need’ in turn drives lexical organisation with high-diversity words being easier to access.

However, Hollis (2020, Experiment 1) suggests that count-based diversity is a poor measure of need, as it does not capture variability across the contexts in which a word is used. They compared traditional count-based diversity, calculated over corpora with the structure of the materials intact, with a new measure (CDrand), calculated over documents of the same length but with the content words randomised, such that the only difference between the two metrics was whether they were calculated over topically coherent documents. They found that, although traditional count-based diversity explained more variance in lexical decision reaction times than word frequency, it did not explain significantly more variance than CDrand. This suggests that any additional variance explained by count-based diversity over and above that of word frequency is not a result of it capturing differences in the contextual usage of a word. Based on this, Hollis (2020) argued that count-based diversity calculated over the traditional window size is not a fundamentally different construct from word frequency and is a poor measure of how likely it is for a word to be needed in a new context. This then questions whether the effects of count-based diversity reviewed in the behavioural studies section stem from these metrics capturing diversity in how words are used, or because they capture frequency somewhat differently to existing frequency measures.

For count-based diversity to better capture variability in the contexts in which a word is used, Hollis (2020) suggests that it should be calculated over smaller window sizes, as long documents may span multiple topics, and dividing them into smaller segments may better capture shifts across topics. Supporting this, Experiment 2 found that count-based diversity accounted for most variance in lexical decision reaction times when calculated over relatively small window sizes of 4–32 words (the largest window sizes considered were 1,024 words wide or an entire document regardless of length). Experiment 2 also included another measure of diversity, word burstiness (see Table 2). Words have a high ‘burstiness’ score if they occur multiple times within a context window. Burstiness also accounted for most variance in lexical decision reaction times when calculated over windows of 4–32 words and accounted for more variance than count-based diversity across all window sizes and corpora. This suggests that measures of diversity that also take word frequency into account may provide more valid estimates of the accessibility of words. However, in Experiment 3, when diversity was calculated over Spanish, Dutch, and French Wikipedia and Subtitles corpora, the results were inconsistent as to the optimum window size and whether burstiness accounted for more variance than count-based diversity. Hollis suggested that the effects of diversity, whether count-based or burstiness, may be specific to the English language. However, many of the language corpora were small and may therefore not have provided good estimates of diversity. Gimenes and New (2016) showed that, once English translations are removed, the non-English-language corpora are substantially smaller than their English counterparts, which can lead to unpredictable diversity effects, as discussed further in the corpus validations section. Taken together, these studies indicate that the validity of count-based diversity metrics depends upon window and corpus size, and also raise questions as to whether these metrics are optimal measures of the diversity of a word’s usage.

In contrast, Johns et al. (2020) and Johns (2021b) found that calculating count-based diversity metrics over larger contextual units explains greater variance in behavioural data. Johns et al. (2020) showed that a word’s book and author prevalence (see Table 2) derived from a Google Books corpus were a substantially better fit to lexical decision and word naming accuracy and word prevalence ratings than traditional count-based diversity (context defined as 20 consecutive sentences), with author prevalence being the strongest predictor in all cases. Although, this was not the case for lexical decision or word naming times. Johns (2021b) compared three metrics computed from a Reddit corpus (see Table 2) in which diversity was calculated across individual comments (traditional count-based diversity), all comments of a user (user count-based diversity), or all comments in a subreddit (discourse count-based diversity). Across mega-study datasets of lexical decision accuracy and reaction times, word naming accuracy and reaction times, and word prevalence ratings and response times, user and discourse diversity accounted for significant variance over and above traditional count-based diversity, with discourse diversity accounting for most unique variance overall. The superiority of discourse diversity was also reported by Senaldi et al. (2022) for subjective familiarity ratings of words and multiword English idiomatic phrases.

Taken together, in contrast to Hollis (2020), this set of experiments indicates that count-based metrics calculated over large units of measurement, such as the collective works of an author or all comments in a subreddit, are better predictors of word-form processing than count-based metrics calculated over traditional window sizes. This may be because, rather than attempting to capture whether the usage of a word changes across topics, these large-scale metrics instead capture the likelihood that people will be familiar with a word (Johns et al., 2020). Furthermore, these metrics suggest that social usage of a word may contribute to lexical organisation. Specifically, Johns et al. (2021b) suggest that words or phrases used across discourses are those that are most likely to be ‘needed’ when conversing with a new person and therefore have the strongest representations in memory. However, it is difficult to draw any firm conclusions as these large-scale metrics have not been directly compared to those calculated over smaller window sizes, so it is unclear which best account for behavioural data. Furthermore, Hollis calls into question whether count-based metrics in general truly capture information about the linguistic structure of the materials from which they are derived. If the large-scale metrics are indeed capturing something additional about social language usage, we would expect them to out-perform their equivalents calculated over randomised documents.

##### Computational metrics

One general limitation of count-based models is that they do not necessarily capture variability in the semantic content of the contexts in which words occur. Predict and SDM-derived diversity metrics have sought to account for this.

Hollis (2017) compared count-based diversity with measures of diversity extracted from two predict models: CBOW and skip-gram (see Table 2). These models generate a context vector (also called an embedding) that represents a word meaning through predicting a word’s use across a corpus of text. In Experiment 1, when all semantic features in the embedding were weighted equally, count-based diversity explained more variance in lexical decision times than the diversity values derived from both predict models. However, in Experiment 2, when the weighting of the semantic features was allowed to vary, diversity as derived from embeddings generated by the predict models accounted for significantly more variance than count-based diversity. However, count-based diversity and word frequency still accounted for significant unique variance in the data, suggesting that the pattern of a word’s use across a corpus, the number of contexts in which a word appears, and the number of times a word occurs all contribute to lexical organisation.

In contrast, in the first iteration of SDM-derived diversity (see Table 2), Jones et al. (2012) found that this metric explained all variance in lexical decision and word naming times accounted for by traditional count-based diversity and word frequency, as well as additional unique variance. This suggests that the semantic structure of the contexts in which a word is experienced is more important in lexical organisation than frequency or count-based diversity.

Johns et al. (2020) then derived SDM versions of their book prevalence and author prevalence metrics and compared these with their original count-based versions. Low-diversity words are used in the same way across multiple books, or by multiple authors, whereas high-diversity words are used in more varied ways across books or authors. SDM book and author prevalence metrics accounted for more variance in lexical decision and word naming accuracy and latencies and word prevalence ratings than their count-based alternatives. Interestingly, traditional SDM-derived diversity (context considered as a moving window of 20 sentences) also accounted for very little variance when SDM book and author prevalence metrics were included in the analysis. SDM author prevalence accounted for the most unique variance across datasets.

Johns (2021b) also computed SDM versions of the user and discourse contextual diversity metrics from the Reddit corpus. They did this at two levels; the word level, which computes diversity over the same context units as their count-based equivalents (single comment or subreddit), and the population level, which takes all comments produced by a user within a subreddit as a context. The key difference between the user semantic diversity and discourse SDM-derived diversity metrics calculated at the population level is how the model updates the semantic distinctiveness value for a word. For user semantic diversity, the semantic distinctiveness value for a word is updated when that word is used in a new subreddit by the same user. For discourse semantic diversity, the semantic distinctiveness value for a word is updated when that word is used by a new user within the same subreddit. These measures aim to capture diversity in language use across users, with user SDM-derived diversity at the population level measuring how consistently a word is used within users across discourses, and discourse SDM-derived diversity, measuring how consistently words are used within discourses across users. User and discourse SDM diversity were found to account for more variance in lexical decision, word naming, and word prevalence data than word frequency and count-based diversity^22^, and these increases in variance explained were greater than when using their count-based equivalents. Models trained using population-level representations accounted for more variance than those trained using word-level representations.

With this user SDM diversity metric calculated at the population level, Johns and Jones (2022) examined how manipulating the materials the model is trained on affects the resultant metric. When trained on a smaller sample of the Reddit corpus, the model accounted for less variance across all datasets than when trained on the full corpus; however, it still accounted for significantly more unique variance than word frequency. In addition, the difference in variance explained by word frequency calculated across both the full and sampled corpora was not significant. This further demonstrates that metrics that rely on distributional information improve when more word co-occurrence data is available. This in turn suggests that new contextual experiences are essential for updating of word representations, and that, after a point, repetition of a word does not influence lexical strength. They then investigated how randomising the training materials affected the resultant metrics, akin to Hollis (2020). Models were trained on materials that retained 100%, 75%, 50%, 25%, or 0% of user comments, with the remainder randomly replaced with other users’ comments. Unlike results from Hollis, as the corpora were increasingly randomised, the amount of unique variance explained by the SDM systematically decreased, whereas the amount of variance explained by word frequency increased. Randomising comment patterns turns the corpora into samples of average language meaning that distinctiveness in language use between individuals is lost. These results therefore suggest that the advantage for user SDM-diversity over word frequency is due to the semantic coherency of the training corpus. Specifically, the way the model captures the unique communication patterns of individuals.

A further conclusion drawn by Hollis (2020) was that count-based measures performed best over smaller window sizes. Johns and Jones (2022) investigated whether this was true for the user SDM population level metric derived from the reddit corpus. They reduced the context size the metric was calculated over by including fewer user comments in the training materials and reducing the number of discourses over which the model was trained (100% to 10% in steps of 10%). They found that the user SDM metric explained less variance in lexical decision, word naming, and word prevalence data for smaller context sizes and when trained across fewer discourses, however it still explained more variance than word frequency. They also showed that when only the most distinctive contexts were used, a count-based version of the metric, SDM_Count, fit the behavioural data as well as the user SDM population level metric. This further confirms that that the updating of lexical representations is most influenced by distinctive contexts, with repeated or redundant contexts having little impact.

Using the count-based and SDM-derived user and discourse diversity metrics at both the word and population representation level, Senaldi et al. (2022) found that user SDM diversity at the population level accounted for most variance in single word familiarity ratings and that word frequency no longer explained significant variance, in line with the results for single word recognition data. They also examined idiom familiarity ratings, finding that discourse SDM diversity at the population level accounted for most variance, but that frequency still accounted for unique variance. This suggests that different mechanisms may underly the storage of single versus multiword expressions.

Taken together, these results suggest that the social structure of language use contributes to lexical organisation and that structuring corpus training materials to capture these patterns of usage, in addition to semantic information (e.g., topic overlap), leads to diversity metrics that better capture human behaviour. The population-based user SDM was most successful across outcome measures of single word processing. This suggests that the diversity of use across discourses is the primary determinant of lexical strength, presumably because this best captures whether a word is likely to be needed in a new discourse and/or when talking to a new person. On the other hand, when words are used by a small subset of the population in strongly defined discourses they have weaker representations as they are unlikely to be used outside of this specific setting. However, for outcome measures relating to multiword expressions, the diversity of use of within discourses seems to determine their lexical strength, combined with their frequency of usage.

##### Comparisons with polysemy

The final computational modelling study that met our inclusion criteria took a very different approach to exploring the impact of diversity metrics on word-form processing. Cevoli et al. (2021) focused on the relationship between diversity and polysemy, the number of senses a word has based on the structure of dictionary entries (see Rodd, 2020, for further discussion of the effects of polysemy on lexical access). Most diversity metrics used in the computational modelling experiments discussed above were derived using the SDM (Jones et al., 2012). However, Cevoli et al. focused on LSA-derived diversity (Hoffman et al., 2013), which is the most common computational metric used in behavioural studies (Fig. 3) and was originally intended as a graded measure of polysemy. In a series of three simulations, Cevoli et al. investigated whether the facilitatory effects of polysemy on lexical decision accuracy and reaction times could be attributed to variations in LSA-derived diversity. Rather than using lexical decision data from megastudy datasets, Cevoli et al. used data from two experimental studies of ambiguity effects on word-form recognition (Armstrong & Plaut, 2016; Rodd et al., 2002). Both studies demonstrated that word recognition was faster for words with multiple related senses (polysemes) compared to few senses, but slower for words with multiple meanings (homonyms) compared to those with a single meaning. According to Hoffman et al. (2013), words with fewer senses or one meaning should be lower in LSA-derived diversity than those with many senses and multiple meanings. However, Cevoli et al. found that LSA-derived diversity did not differ between words with few senses/meanings and those with multiple senses/meanings, suggesting that this metric does not appropriately capture variations in polysemy and, as such, the word recognition advantage for polysemous words cannot be attributed to LSA-derived diversity. Cevoli et al. instead suggested that LSA-derived diversity captures general information about the spread of topics in which a word is used but is insensitive to shifts in meaning. This is in line with the findings of Taylor et al. (2022), who demonstrated that there was no difference in how easy it was for participants to think of a context for high and low LSA-derived diversity words.

In response, Hoffman et al. (2021) argued that Cevoli et al.’s (2021) results were due to the way they computed the LSA-derived metric, which used scaled as opposed to unscaled vectors. This approach leads to vectors with larger singular values being assigned more weight, meaning they ‘count more’ when cosines between vectors are computed (Hoffman et al., 2021). This gives a disproportionate weighting to the first two dimensions in the semantic space, which are strongly correlated with word frequency. Hoffman et al. argued that this means that the metric calculated by Cevoli et al. overestimates the importance of frequency, which may be detrimental when determining semantic relationships between the contexts. Hoffman et al. replicated Cevoli et al.’s analyses comparing their operationalisation of LSA-derived diversity (scaled vectors) and the original Hoffman et al. (2013) metric (unscaled vectors). For both Rodd et al.’s (2002) and Armstrong and Plaut’s (2016) data, words higher in LSA-derived diversity had a higher number of senses using the Hoffman et al. (2013) but not the Cevoli et al. metric. However, neither the Hoffman et al. (2013) nor the Cevoli et al. metric predicted number of meanings (i.e., homonymy) for either dataset. Taken together, these studies suggest that although LSA-derived diversity is capturing something about the diversity of contexts in which a word appears, it is unclear whether this provides a graded measure of polysemy as originally intended.

#### Other outcome measures

##### Recognition memory

Johns (2021a) examined the performance of diversity metrics in explaining variance in recognition memory data for written words. Across several count-based and SDM-derived diversity measures (calculated according to Johns, 2021b), high diversity was associated with better recognition memory, with higher hit rates and fewer false alarms for high compared to low-diversity words. These results contradict behavioural studies that reported a consistent low-diversity advantage for recognition memory (e.g., Cook et al., 2006), again suggesting that this low-diversity advantage may be a product of the stimuli used (Steyvers & Malmberg, 2003) rather than a true diversity effect. Johns also performed regression analyses to determine which metrics explained most variance in performance. Traditional count-based diversity accounted for significantly more variance than word frequency, and discourse followed by user count-based diversity accounted for more variance than traditional count-based diversity. User and discourse SDM diversity at the population-based level explained more variance than their count-based equivalents, however SDM metrics using word-based representations did not. Overall, population-based discourse SDM-derived diversity explained most variance in the recognition memory data.

These results highlight that no one metric best accounts for performance across all datasets. For recognition memory and idiom familiarity, discourse SDM diversity (population level) was the best predictor of behaviour, however, for single word-form processing, user SDM-derived diversity (population level) explained most variance. Johns (2021a) suggests that this demonstrates that multiple types of contextual information can be contained in a word’s lexical representation, and that these are accessed differently according to task demands. In this example, accessibility of isolated word forms may be driven by how a word is used across individuals, whereas memorability of a word may be tied to how a word is used across discourses.

##### Age of acquisition

Two experiments investigated how diversity metrics relate to age of acquisition for different word types. Hills et al. (2010) operationalised diversity as the number of unique words a word co-occurs with in child-directed speech, using the American section of the CHILDES corpus (MacWhinney, 2014). Experiment 1 found that higher diversity was associated with earlier age of acquisition, and that this was most predictive when calculated over a relatively small window size of around five words (2–100-word window sizes were examined). The effect was strongest for nouns. Experiment 2 investigated the mechanisms by which diversity facilitated acquisition. They constructed networks from words in the McArthur-Bates Communicative Developmental Inventory (CDI), a large-scale study of children’s productive vocabularies from 16–30 months (Dale & Fenson, 1996), and determined the structural properties of the new words that were added each month. For nouns, the number of words the word was semantically related to in the network determined whether that noun was added to the network. However, for function words and verbs, the total number of words the word was connected to determined whether it was added to the network.

The results of Hills et al. (2010) suggest that the structure of caregiver speech influences when words are acquired, with high-diversity words acquired earlier. Some behavioural studies also showed that words high in LSA-derived diversity were acquired earlier (Reggin et al., 2021). Taken together these findings suggest that diversity in both speech and written language predicts when words are acquired. However, the mechanisms underlying this may differ across word classes.

#### Conclusions

Concerning the effects of count-based diversity on word-form processing, the modelling work shows an apparent contradiction regarding the optimal window size over which diversity should be calculated. Hollis (2020) suggests that count-based diversity best accounts for behavioural data when calculated over smaller window sizes, which may capture shifts in topic within a document. However, other work reports that count-based diversity best accounts for behaviour when calculated over much larger units of measurement, which may capture patterns of usage across discourses or individuals (Johns et al., 2020; Johns, 2021b; Senaldi et al., 2022). These discrepant results may indicate that these metrics tap two different constructs. Hollis’s (2017, 2020) diversity and burstiness metrics can be conceptualised as alternative measures of frequency that capture how often words occur within a corpus, but not necessarily how likely it is that participants have encountered a word. On the other hand, prevalence-based metrics that account for usage across discourses or individuals (Johns et al., 2020; Johns, 2021a, 2021b; Senaldi et al., 2022) give a better indication of the likelihood of individuals experiencing a word (or phrase) in their day-to-day lives, and therefore how likely it is to be ‘needed’ on future encounters.

A further clear conclusion is that computational metrics that account for the semantic content of the documents in which a word appears explain more variance in behavioural data than count-based metrics. This suggests that appearing in more semantically unique contexts leads to stronger updating of lexical representations than appearing in contexts with a high level of semantic overlap. SDM-derived metrics explain most variance when calculated over larger contextual units that capture patterns of language use across individuals (e.g., authors or forum users) or discourses (e.g., books or subreddits). This suggests that the complex social structures of language play an important role in lexical organisation. As such, these studies demonstrate that our lexical representations capture multiple sources of diversity, including both the semantic structure and communicative patterns of language, and that this may help us to predict which words are most likely to be needed in future contexts.

Finally, predict models trained over small window sizes also outperform count-based metrics, suggesting that patterns of local word cooccurrence may also shape lexical representations (Hollis, 2017). Local environment also appears to have the most influence on infant language development (Hills et al., 2010). As no direct comparison has been made between all these models for the same datasets we are not able to draw strong conclusions as to whether local or global contexts have the most influence on developing lexical representations. One intriguing possibility is that in the early stages of acquisition we rely on local contextual information to extract word meanings, but that global information has a greater impact on lexical representations as linguistic experience progresses. However, one theoretical issue highlighted by these studies is whether count-based metrics are better conceptualised as modified frequency measures. This argument was made by Hollis (2020) based on analyses suggesting that count-based metrics are not sensitive to randomisation of the topical content of the contexts across which they are calculated. Jones et al. (2017) propose that the improvement shown for count-based metrics over traditional frequency measures is because they capture information about the *likelihood* of a word being encountered, rather than diversity of a word’s usage across contexts. However, in addition to accounting for more variance in megastudy datasets of word-form processing than count-based metrics, SDM-derived metrics, particularly those accounting for patterns of social usage, are sensitive to randomisation of the source materials (Johns & Jones, 2022; Johns, 2021a, 2021b). This suggests that they successfully capture variations in linguistic usage of a word, and therefore be a more optimal choice of metric for researchers investigating how the diversity of linguistic contexts in which a word is experienced influences lexical processing.

### Corpus validations

Fourteen of the experiments included in this review were categorised as Corpus Validations. Unlike the experiments included in the other two sections, these do not directly set out to investigate diversity effects on behaviour, or to compare the variance explained by different diversity metrics. The primary aim of these experiments is the creation of corpora, and the provision of norms for different lexical variables, including diversity, as a resource for future researchers. These experiments provide insight into whether contextual diversity effects are consistent over corpora constructed from different materials and in different languages.

#### Operations and terminology of diversity

All 14 experiments operationalised diversity using a count-based metric in which the contextual unit was an entire document (Adelman et al., 2006). This was termed contextual diversity in all but one instance where it was termed dispersion (Pham et al., 2019).

#### Word form processing

All corpora were validated using word-form processing tasks, with lexical decision used for every corpus and word naming used in two instances (Brysbaert & New, 2009; Cai & Brysbaert, 2010). Details of corpora contents and creation are provided in the full database of included experiments available on the OSF (https://osf.io/ys6ne).

Only five experiments reported the direction of the diversity effect, with all five using lexical decision and one additionally using word naming. Consistent with the effects reported in the earlier sections, all reported a high-diversity advantage, with words appearing in more documents responded to more accurately and/or faster than those appearing in fewer documents. One of these experiments was conducted using English (van Heuven et al., 2014), two using Chinese (Cai & Brysbaert, 2010), and two using Hindi (Verma et al., 2022), indicating that the high-diversity advantage on form processing is stable across languages.

All experiments considered whether contextual diversity explains significantly more variance in word-form processing than word frequency and most were consistent with Adelman et al. (2006), such that count-based diversity explained more variance than word frequency in lexical decision accuracy and/or reaction times in Catalan (Boada et al., 2020), Dutch (Keuleers et al., 2010), English (Brysbaert & New, 2009; van Heuven et al., 2014), Polish (Mandera et al., 2015), Portuguese (Soares et al., 2015), and Vietnamese (Pham et al., 2019). In contrast, for Chinese, Cai and Brysbaert (2010) found that although subtitle count-based diversity explained more variance in lexical decision times to Chinese two-character words, word frequency explained more variance in single-character naming latencies.

In Hindi, Verma et al. (2022) found that diversity calculated over a corpus of websites and newspaper articles did not explain more variance in lexical decision accuracy and reaction times than word frequency. Gimenes and New (2016) created three corpora from Twitter posts, blog posts, and web-based news sites for five languages: Chinese, Dutch, French, English, and Malay. Diversity did not account for significantly more variance in lexical decision reaction times than word frequency for English, Malay, or Chinese in any of the three corpora. In Dutch, word frequency accounted for more variance than diversity across all three corpora and, in French, diversity only accounted for significantly more variance than word frequency in the blog corpus. Given the consistent high-diversity advantage on word-recognition tasks shown throughout this review, these findings may reflect limitations of the corpora. This is supported by Gimenes and New, who suggest that context length may play a role and acknowledge that, due to copyright reasons, most of their documents consisted of only one to three sentences. Count-based diversity differs from word frequency as it ignores repetitions of a word in the same context. Words are less likely to be repeated within shorter documents and thus diversity and frequency values are far more similar in short twitter/blog posts than when diversity is calculated over an entire film or television programme. In other words, as window size gets smaller, a word’s frequency and diversity values become more similar, until at a window size of one they are identical. This underscores the idea that count-based diversity and word frequency may be the same construct just calculated over different window sizes (Hollis, 2020).

In summary, the results from corpus validations are consistent with both the behavioural and computational modelling studies in showing that count-based diversity is generally superior to word frequency in explaining variance in word-form recognition data. Additionally, they show that this advantage is broadly consistent across languages. However, the explanatory advantage of diversity over frequency can be affected by the type and size of the documents over which it is calculated. Diversity effects are strongest when count-based diversity is calculated across large corpora consisting of large contextual units. This is exemplified by the fact that effects are strongest and most consistent for subtitles corpora, where the contextual unit is the entire set of subtitles for a film or television show. For corpora where the contextual units are smaller (e.g., a single tweet), word frequency and count-based diversity become more similar, attenuating the diversity advantage.

## Discussion

This review provides a comprehensive overview of studies that assess the effects of contextual diversity on lexical processing, summarising evidence from 86 articles including 145 experiments. These experiments were conducted in 18 different countries in nine different natural languages. Three distinct types of experiment were identified: behavioural studies, computational modelling studies, and corpus validations, forming three review sections. Within these sections, we identified the different ways in which diversity has been defined and operationalised across studies, before discussing diversity effects across tasks with shared methodologies. This allowed us to identify common patterns of results, map inconsistencies and knowledge gaps, and provide recommendations for future research.

### Summary of evidence

This review has answered several research questions as summarised below.

#### RQs 1 and 2. How has diversity been defined and operationalised in the literature?

This review has highlighted the wide range of ways that diversity has been operationalised across studies. Unfortunately, inconsistent terminology has been used to refer to these operationalisations (e.g., LSA-derived diversity, as first operationalised in Hoffman et al., 2013, has been termed *ambiguity*, *contextual distinctiveness*, *number of senses*, *semantic distinctiveness*, and *semantic diversity*; Fig. 3). A key contribution of this review has been to identify common methodologies across these operationalisations, enabling us to categorise experiments according to four overarching metric types (Table 2). This allowed us to integrate findings across experiments using similar metrics when answering subsequent review questions. It will also allow researchers to easily identify how their results fit within the field and to better understand patterns of results in future research.

#### RQ3. What outcome measures have been used to investigate the effects of diversity on word-form processing?

Most experiments focused on word-form processing (85 out of 145 experiments). Within these, lexical decision was the most frequently used task, with standard visual lexical decision used 51 times, and variants of this task used nine times. Word naming was used second most frequently in 22 experiments. Five other word-form processing outcome measures were each only used once (see Table 3).

#### RQs 4 and 7. Does diversity affect word-form processing, and what factors modulate these effects?

The behavioural experiments, computational modelling studies, and corpus validations all show a consistent advantage for high- over low-diversity words on tasks of word-form processing, across metrics (though most have used count-based metrics; Fig. 4). The majority of modelling and corpus studies also show that count-based diversity explains more variance in word-form processing than word frequency. These findings support the idea that high diversity speeds lexical access in line with the predictions of Nation (2017) and Perfetti and Hart (2002).

Several researchers have developed computational and composite diversity metrics that explicitly account for semantic variation in the contexts in which a word is used (e.g., Burch et al., 2016; Hoffman et al., 2013; Jones et al., 2012; McDonald & Shillcock, 2001; Musz & Thompson-Schill, 2015). These have not been used as extensively as count-based metrics in behavioural experiments investigating word-form processing; however, computational modelling studies consistently show that SDM-derived metrics, which compute diversity based on the proportion of overlapping words in the contexts in which a word occurs, explain more variance in word-form processing than count-based metrics (Johns & Jones, 2022; Johns et al., 2020; Johns, 2021b; Jones et al., 2012). These studies provide compelling evidence that experiencing a word in a diverse range of contexts benefits subsequent form-based processing.

A further interesting finding from the modelling studies was the efficacy of ‘prevalence-based’ diversity metrics in explaining word-form processing performance. These are extracted over larger contextual units, such as entire discourses, and aim to capture social patterns of a word 's usage (e.g., whether a word is used by few individuals in specific discourses or by a diverse set of individuals over many discourses). These consistently outperform the same metrics calculated over the traditional definition of a context (i.e., a single document), suggesting that lexical organisation is sensitive to how groups of individuals use words across contexts as well as the semantic content of these contexts.

In addition, we reviewed how these effects on word-form processing might be modulated by item- or participant-level factors. The only clear item-level effect is that word frequency interacts with SDM- and LSA-derived diversity, such that the high-diversity advantage for lexical decision and word naming is greatest for high-frequency words (Chapman & Martin, 2021; Hamrick & Pandza, 2020; Hsiao et al., 2020; Jones et al., 2012). This suggests that repetitions of a word in more distinctive (non-overlapping) contexts lead to stronger updating of lexical representations than experiencing words in semantically similar contexts. This is supported by the computational modelling studies, which showed that when only the most distinctive contexts were used, count-based models explained similar variance in behavioural data to the SDM-derived metrics (Johns & Jones, 2022). In other words, these studies suggest that it is the distinctiveness of repetitions that drives lexical updating, rather than repetition alone. In terms of participant-level factors, there is some evidence to suggest that diversity effects vary depending on linguistic experience (e.g., bilingualism, age, length of language learning); however, more research is needed to draw firm conclusions.

To summarise, across behavioural experiments, computational modelling studies, and corpus validations there is a consistent advantage for diversity measures over traditional word frequency measures in explaining performance on word-form tasks. As suggested by Adelman et al. (2006) and Johns (2021b), this may be because measures of diversity capture whether a word is likely to be ‘needed’ on a future encounter. This review highlighted several aspects of diversity that may contribute to ‘need’:

1. The number of documents in which a word appears. Words that appear in more documents are more likely to be needed when encountering a new document than those restricted to few documents.
2. The semantic content of those documents. Words that appear in a variety of semantic contexts are more likely to be needed in a new context than those appearing in specific contexts.
3. The pattern of word use within social discourse. Words that are used consistently across people are more likely to be needed when interacting with someone new than those that are only used by a specific subset of the population.

Taken together, this suggests that, over time, individuals integrate information about how often a word is used, the semantic context in which a word is used, and how different people use a word, into their lexical representations. This then gives an indication of how likely a word is to be needed, and thus how accessible that word form is, on future encounters.

#### RQ5. What outcome measures have been used to investigate the effects of diversity on word-meaning processing?

Far fewer experiments have investigated the effects of diversity on word-meaning processing with almost all employing a different outcome measure. The only tasks used more than once were concreteness decision, semantic relatedness decision, and creative word generation (see Table 4).

#### RQs 6 and 7. Does diversity affect word-meaning processing, and what factors modulate these effects?

**Fig. 8:**
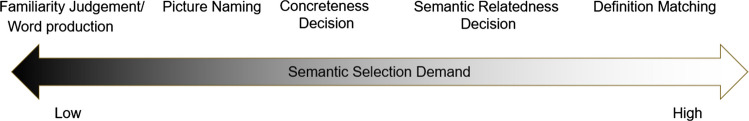
Illustrative example of the degree to which the tasks discussed in this review require semantic selection

In contrast to form processing, word-meaning processing has largely been investigated with LSA-derived metrics that account for semantic variation in the contexts in which words occur (Hoffman & Woollams, 2015; Hoffman et al., 2013; Hsiao & Nation, 2018; Hsiao et al., 2020; Taylor et al., 2022). Unlike the consistent high-diversity advantage seen on tasks of word-form processing, the influence of diversity on word-meaning processing is more mixed, depending on both task and metric type. This variation in diversity effects across tasks may reflect the extent to which they require participants to select a single, precise word meaning (as illustrated in Fig. 8).

For picture naming, semantic selection is not required as responses are constrained by the visual features of the stimuli, and null effects of diversity are typical on this task. For tasks that require general semantic judgements but not selection of a precise meaning, such as concreteness decision, there generally seems to be high-diversity advantage, at least when operationalised using count-based metrics. This parallels results from the form processing literature, where familiarity judgement/production tasks also do not require precise (or indeed any) meaning selection. On the other hand, when tasks require selection of a precise meaning, such as semantic relatedness judgement and to an even greater extent cross-modal definition matching, there is a low-diversity advantage. This may be because such tasks invoke competition between the different contexts (or interpretations) associated with the meaning of these words. However, we make these suggestions tentatively, since research has favoured certain metrics for certain tasks making it difficult to separate the effects of these two variables.

This review also highlights that diversity may be confounded with polysemy (i.e., variability in a word’s meaning; see Hulme et al., 2023), with only four experiments, all using count-based metrics, explicitly controlling for dictionary-based polysemy. The confound is particularly likely with computational metrics, which capture variability in the semantic contexts in which words occur. Indeed, the original Hoffman et al. (2013) LSA-derived metric was intended as a graded measure of polysemy on the basis that “words that appear in a wide range of contexts on diverse topics are more variable in meaning than those that appear in a restricted set of similar contexts” (p. 1). However, whilst dictionary-based polysemy metrics are clearly limited (Beekhuizen et al., 2021) it is not a given that contextual diversity and polysemy are equivalent. Instead, they may reflect distinct lexical characteristics that could usefully be captured by different metrics. In particular, it is currently unclear whether the observed effects of diversity arise solely as a consequence of a word’s contextual history, or whether they also reflect the extent to which the precise meaning of a word varies across these different contexts. This potential confound may contribute to the mixed results on tasks of word-meaning processing. For example, precise meaning judgements may be more difficult for high- than low-diversity words not only due to appearing in more varied contexts and thus having more flexible representations, but also because they require participants to select a single sense of the word, supressing information related to irrelevant senses (Rodd, 2020). Further work is needed to determine the extent to which these two variables are theoretically and practically dissociable, and then to disentangle their effects on different tasks.

#### RQ8. What other behavioural outcome measures have been used to investigate the effects of diversity on lexical processing?

We identified 58 experiments that contained at least one outcome measure that could not be straightforwardly categorised as a measure of either word-form or word-meaning processing.

A substantial amount of work has been conducted on the effect of diversity on memory (39 experiments; see Table 5), primarily using count-based diversity metrics. This literature has largely been overlooked by research on lexical processing. In addition, eight experiments used eye-tracking, which produces outcome measure that are likely to be sensitive to both form- and meaning-based aspects of lexical processing. All other outcome measures were used infrequently, providing insufficient evidence on which to draw firm conclusions (see supplementary materials: https://osf.io/9h7ac).

#### RQ9. Does diversity influence other behavioural outcome measures?

In contrast to form-processing tasks, there was generally a low-diversity advantage in memory tasks, with some evidence that this effect might interact with frequency and with language experience. However, the validity of these effects is uncertain as it may be restricted to the set of stimuli developed by Steyvers and Malmberg (2003), which were not properly equated on word frequency, nor on other dimensions such as concreteness and age of acquisition (Neath et al., 2022). In the few cases where better matched stimuli have been used, diversity effects are less clear (Aka et al., 202; Guitard et al., 2019; Neath et al., 2022). Furthermore, computational modelling studies using megastudy datasets report the opposite effect—that high diversity, both count-based and SDM-derived, benefits recognition memory (Johns, 2021a).

The results of the eye-tracking studies were broadly consistent with the single-word processing results (Chen et al., 2017a, 2017b; Pagán et al., 2020; Plummer et al., 2014). There was a high count-based diversity advantage on both early- and late-stage processing measures, indicating that diversity also facilitates identification and integration of words in sentence contexts.

### Future directions

**Fig. 9:**
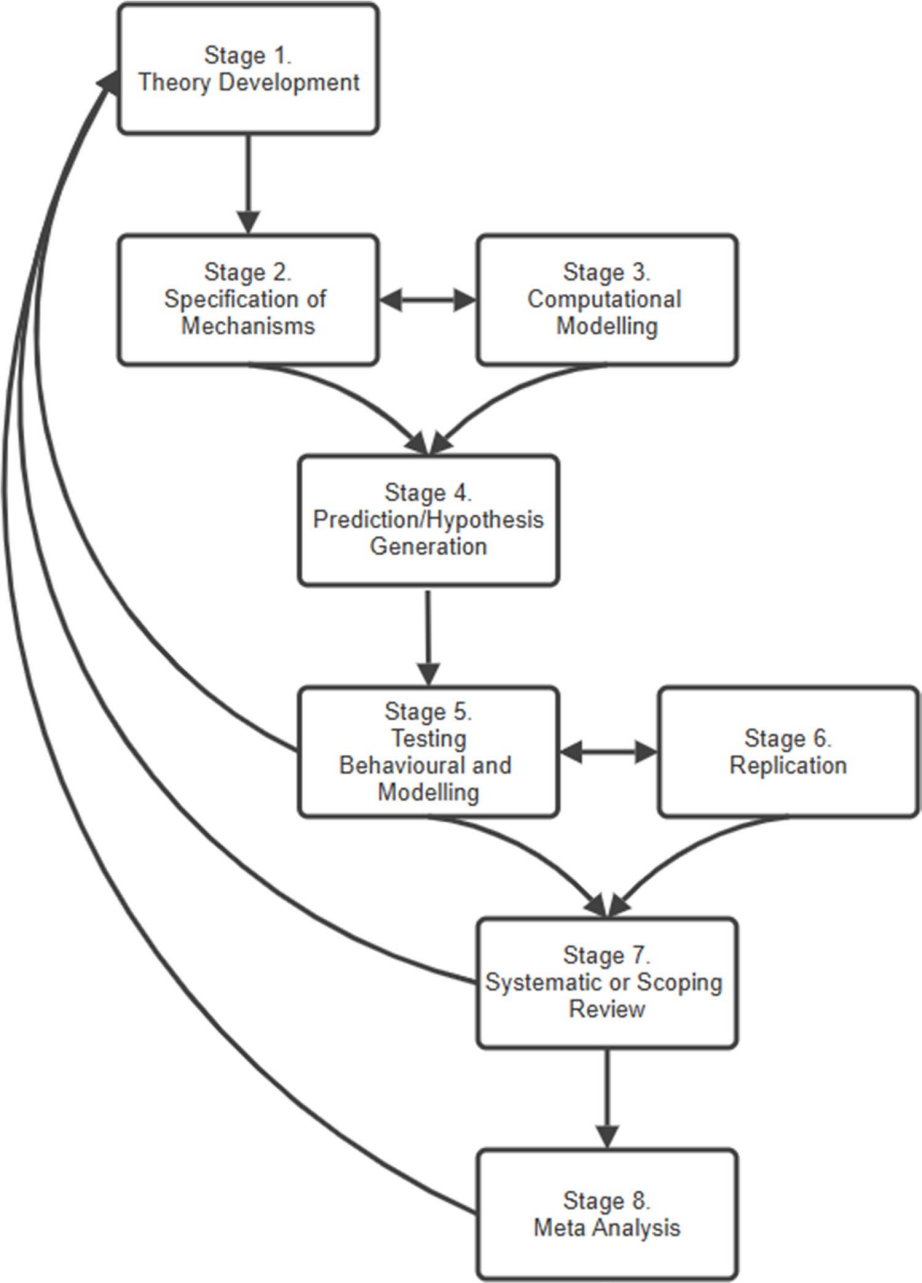
The proposed research cycle. We suggest that computational modelling can potentially play an important role at two stages of the research cycle. First, the process of implementing any specific theories/mechanisms within a particular computational framework requires researchers to be more precise and concrete about their claims and allows for increased certainty about any specific predictions/hypotheses made about human behaviour (e.g., Hills et al., 2010, Stage 3). In addition, by directly comparing the performance of implemented computational models under a range of conditions can provide valuable empirical data that can feed back into the development of theories/mechanisms (Stage 5). The majority of computational modelling studies reviewed here contribute to this latter stage (e.g., Hollis, 2020; Johns & Jones, 2022).

Conducting this review has enabled us to determine how each subfield of research on contextual diversity contributes to the stages of the ‘research cycle’ (Oberauer & Lewandowsky, 2019; Scheel et al., 2021) and to suggest at which stages future research should focus its efforts (Fig. 9). For example, some subfields may require more data, others may be at a point where a meta-analysis would be helpful to integrate heterogeneous findings, and some might benefit from computational modelling to explore potential mechanisms in greater depth. By integrating experimental evidence across subfields, evaluating how well the current theories align with this evidence, and identifying what would most benefit each subfield, this review will support future research on contextual diversity to advance in a structured way.

Stage 1 in the research cycle is to propose a general theory for why contextual diversity should influence lexical processing. Research on word-form and word-meaning processing has largely been situated within the Lexical Quality (Perfetti & Hart, 2002) and Lexical Legacy (Nation, 2017) hypotheses, whereas research on memory has often been interpreted within fan theory (e.g., Cook et al., 2006). These general theories make different predictions for how diversity should influence behaviour, with the former proposing a high-diversity advantage for word-form processing and meaning generalisation, and the latter a low-diversity advantage for item recognition/recall. Work is clearly needed at Stages 2 and 3 in the research cycle to develop these general theories into mechanisms that are specified in greater detail (ideally implemented within computational models), for how and why diversity influences behaviour and whether this does indeed differ across outcome measures.

Some more specific mechanisms for how diversity operates have been proposed (Stage 2). For example, the principle of likely need (Adelman et al., 2006; Anderson & Milson, 1989), suggests that high-diversity words are more likely to be needed in a new context and are therefore more accessible in memory, and Hoffman et al. (2011) suggest that high-diversity words have more variable representations, which are associated with higher levels of baseline activation. Note that both of these propose that diversity should benefit word-form processing. Computational models (Stage 3) are needed to explicitly instantiate these mechanisms and test whether and how they influence different behavioural outcomes. For example, networks could be built that encode the contextual history of a word based on different count-based, computational, and composite metrics, and could then be probed to determine whether high relative to low-diversity words receive more activation and/or differ in interconnectedness. This would then specify more precisely how and why different forms of diversity influence word-form and word-meaning processing, why this might depend on task demands such as degree of meaning selection, how it changes when words are in context vs. isolation, as well influences on memory. Such work would increase precision in theories and proposed mechanisms (Stages 1 and 2), leading to better specified hypotheses (Stage 4) and thus improve all further stages of the research cycle.

As stated in the Introduction, there has been a proliferation of behavioural and modelling experiments testing how contextual diversity influences lexical processing (Stages 5 and 6). A major contribution of this review has been to integrate this research within and across subfields (Stage 7). Despite the large number of studies included in this review (*N* = 145), at this point in time we suggest that the only subfield that is currently suitable for formal meta-analysis (Stage 8) is word-form processing. Here there are a relatively large number of experiments that use similar familiarity and production tasks (*N* = 61), providing suitable datasets to systematically explore the impact of tasks, metrics, and both item- and participant-based variables. In addition, more work is needed using tasks other than lexical decision and word naming (e.g., recognition of degraded stimuli) and with auditorily presented stimuli (Stages 5 and 6). Finally, a full computational comparison of different diversity metrics across the same megastudy datasets of word-form processing would be invaluable (Stage 5).

In contrast, this review shows that the evidence base regarding how diversity affects word-meaning processing is at a far earlier stage of the research cycle. We discovered greater heterogeneity in the tasks being used and far less consistency in results than in studies of word-form processing. More evidence is clearly needed (Stages 5 and 6), both in terms of behavioural and computational modelling experiments, since models of diversity have not yet been applied to data from word-meaning processing tasks. To ensure this subfield advances in a productive manner, future experiments should be designed to test specific predictions regarding the mechanisms that underpin effects of diversity (Stages 2–4). This might be aided by connecting research on how diversity influences word-meaning processing to other fields that have used computational models to understand meaning processing. For example, measures of relatedness derived from distributional semantic models have been shown to predict the perceived meaningfulness of complex phrases (Günther & Marelli, 2016; Vecchi et al., 2011) and novel affixations (Marelli & Baroni, 2015). Whereas metrics discussed in this review have typically examined global co-occurrence patterns (e.g., across an entire document or large window size), work on phrases and affixes has found that local collocation patterns are most predictive or behaviour. Connecting these research areas could therefore help resolve debates as to whether local or global contexts have more influence on developing lexical representations (as raised in the Computational Modelling section) and provide constraints for proposed mechanisms (Stages 2 and 3).

Across all subfields, this review has highlighted the importance of documenting and examining potential item- and participant-level modulators of diversity effects in a consistent way, such that future meta-analyses can assess whether these factors reliably influence performance. Though the majority of studies accounted for word frequency, effects of other factors such as concreteness, age of acquisition, and language experience have not routinely been examined. Perhaps the most important item-level modulator is polysemy. By accounting for semantic variation in the contexts in which words occur, computational metrics capture polysemy to some extent, however very few studies included polysemy as a covariate. More work is needed to tease apart whether diversity exerts an independent influence from polysemy, and how the effect of both variables might depend on task demands such as the degree of semantic selection required.

An additional point to consider is how diversity effects may change when other information is available, for example when the word is embedded in a meaningful sentence or wider linguistic context^23^, or if something is known about the identity of the person using the word. The studies covered in this review have largely focused on single word processing in the absence of other information or prior knowledge. Thinking about the principle of likely need, the presence of additional information may change the need of words, for example, if someone works in the legal profession, they are more likely to need a word like *perjury* than someone who works in construction. In addition, research from other fields has highlighted that word meanings are likely accessed and interpreted online, with the current context in which a word is experienced interacting with contextual history (e.g., Rodd, 2020). Formal modelling (Stages 2–3) followed by behavioural testing (Stages 4–6), in particular eye-tracking during sentence reading, could help in understanding how prior knowledge and environment interact with diversity effects, which would in turn refine theories of how flexibility and adaptability emerge within the lexicon (Stage 1).

### Limitations

Although we conducted this review with strict adherence to the guidelines set out in Peters (2017) there were some limitations. We chose not to include searches of grey literature, information produced on all levels of government, academia, business and industry in electronic and print formats not controlled by commercial publishing (Auger, 2017), which could have contributed to publication biases as nonsignificant results are less likely to be published. We also included only English language sources, so although we included studies from several countries conducted in multiple different languages this may nevertheless have skewed the results. Although we tried to be as comprehensive with our search terms as possible, we may have overlooked some studies that met our inclusion criteria. Finally, the results are only up to date as of June 2022.

### Conclusions

In 2006, Adelman et al. challenged the role of word frequency as an organising principle of the lexicon, instead proposing that the lexicon was organised based on likely need, captured by a metric they termed contextual diversity. Since then, numerous researchers have expanded on these initial findings, showing that diversity effects extend beyond word-form processing, also influencing wider aspects of lexical processing such as semantic judgements and memory.

We have summarised this burgeoning research field, providing a comprehensive overview of the literature investigating the effects of diversity on lexical processing to date. We have shown that, despite differences in terminology and methods, commonalities exist across studies. We proposed a structured research cycle and used this to make specific recommendations about the steps necessary to advance the field and to highlight theoretical questions that are yet to be resolved. As such, this review provides a comprehensive introduction to diversity research for those new to the field. It should also enable more effective research into the role of diversity in lexical processing moving forward by highlighting theoretical gaps, where our knowledge of the mechanisms underlying diversity effects is currently lacking, and helping future researchers make grounded predictions about how different diversity metrics predict performance.

## Data Availability

All datasets generated and experimental materials are publicly available via the Open Science Framework (https://osf.io/bpgr9/).

## Footnotes

1 Adelman et al. (2014): Data were collected by teams in five different countries: UK, USA, Australia, Canada and Singapore. Hashimoto and Egbert (2019): data were collected in the USA and Mexico.

2 Gimenes and New (2016) investigated diversity effects in Chinese, Dutch, French, English, and Malay. Hollis (2020; Experiment 3) investigated diversity effects in Dutch, French, and Spanish.

3 Four metrics fell into this category: Hoffman et al.’s (2013) metric is termed semantic diversity in 28 experiments, but also contextual distinctiveness (two experiments), semantic distinctiveness (one experiment), ambiguity (one experiment), number of senses (one experiment), and H13_SemD in the computational modelling section. Jones et al.’s (2012) metric is termed semantic distinctiveness (two experiments) and semantic diversity (seven experiments). Brysbaert and New's (2009) metric is termed contextual dispersion (three experiments) and contextual diversity (five experiments). Adelman et al.'s (2006) metric is referred to as contextual diversity (42 experiments) and document count (five experiments). Outside of the behavioural studies, multiple names were only given to one other metric—Cevoli et al. (2021) is termed textual diversity and C20_SemD in the computational modelling section.

4 One experiment (Tsang & Zou, 2022) was removed from further analysis as they did not report behavioural data from the lexical decision task. The results for diversity operationalised with a distributional metric (McDonald & Shillcock, 2001) reported in Skalicky et al. (2019) were not included as the authors did not include this metric in their final analysis. Four experiments were not included in the figure as the direction and significance of behavioural results was not reported, only that diversity accounted for a proportion of the variance in the behavioural data (Johns et al., 2012, 2016).

5 Calculated by dividing the probability of knowing a word (i.e., how many test takers respond ‘yes’ to a word) by the probability of not knowing that word (i.e., how many test takers respond ‘no’ to a word).

6 No effect was reported for four experiments using count-based metrics (Guitard et al., 2019, Experiments 4a and 5b; Hsiao & Nation, 2018, Experiment 1; Vergara-Martinez et al., 2017) and two using LSA-derived metrics (Hsiao & Nation, 2018, Experiment 1; Hsiao et al., 2020, Experiment 2). Excepting Hsiao et al. (2020), all experiments that obtained null effects for accuracy found the expected high-diversity advantage for reaction times.

7 Note that Nenadić et al. (2019) did not directly control for word frequency, instead opting to use frequency weighted semantic neighbourhood density, log frequency multiplied by semantic neighbourhood density, to avoid multicollinearity issues between these two variables. This was significantly negatively correlated with reaction times, suggesting that frequency measures have a greater impact on auditory familiarity judgements than semantic diversity.

8 They compared younger and older adults and found a three-way interaction between age, language status, and SDM-derived diversity, with diversity accounting for least variance in the younger monolingual group (*M*_age_ = 21.38 years), then the younger bilingual group (*M*_age_ = 21.68 years), then the older monolinguals (*M*_age_ = 74.06 years), and accounting for most variance in older bilinguals (*M*_age_ = 70.62 years). In addition to these interactions with age and language status,

9 For clarity and consistency across experiments, we chose to rename three of the tasks based on their methodology. Semantic categorisation (Goh et al., 2016; Pexman et al., 2008; Yap et al., 2011) is termed concreteness decision. Synonym judgement (Hoffman et al., 2011, 2013; Hoffman & Woollams, 2015: Experiment 2) is termed semantic relatedness decision, and cross-modal semantic decision (Hsiao et al., 2020) is termed cross-modal definition matching.

10 The results for one experiment were excluded from further analysis as the behavioural results were not reported. Five experiments were excluded from the figure as the direction of the diversity effect was not clearly interpretable. The results for count-based diversity (Hoffman et al., 2013) were not included in the analysis as the authors did not report their findings for this metric. The results for creative word generation (Johnson & Hass, 2022), word association (Van Rensbergen et al., 2015), and context and sentence availability ratings (Taylor et al., 2022) were not included in the figure as the direction of the diversity effect was not clearly interpretable.

11 Words that are linked to the cue but that very few, if any, other people would come up with, so, that if someone else saw the response, they would find it original and/or surprising, but still understand the connection.

12 Dependent measures in Experiment 1 were semantic distance novelty calculated by computing the semantic distance between each prompt noun and the participants’ responses and semantic context search calculated by the semantic distance between all pairwise comparisons of all responses for each prompt noun.

13 Additional measure of semantic context search calculated by clustering responses based on reaction times. Responses were categorised as ‘clusters’ and ‘switches’ using a slope-difference algorithm, with within-cluster responses thought to be more semantically similar than between-cluster responses

14 Data from one experiment (Crossley et al., 2013) were excluded from further analysis as the behavioural results were not reported. Data from eight experiments (Crossley et al., 2013; Heffernan et al., 2018; Hoffman et al., 2014; Linzen et al., 2013; Musz & Thompson-Schill, 2015; Reggin et al., 2021; Tsang & Zou, 2022; Vergara-Martinez et al., 2017) were excluded from the figure as sufficient information was not provided to classify the direction of the diversity effect. Outcome measures from the subjective recognition memory task were also not included in the figure as responses were coded as ‘know’, ‘remember’, ‘lacks familiarity’, or ‘would have remembered’ (Cook et al., 2006; Meeks et al., 2014) and confidence estimates (Papesh et al., 2019) which do not clearly map onto high- or low-diversity advantages.

15 For all tasks, responses were categorised as either hits, correct rejections, or false alarms. From these, multiple outcome measures were calculated; *d′* (*z*-score hit rate − *z*-score false-alarm rate), the mean slope of the *Z*-ROC curves (slope of *z*-score hit rate against *z*-score false-alarm rate), and *d*_a_ (a measure of sensitivity calculated using the vertical intercept, horizontal intercept, and slope of the *Z*-ROC curve). For mouse-tracking, area under curve (the geometric area between the actual trajectory of the mouse and an ideal one) was included as an additional dependent measure.

16 Aue et al. (2018) found a three-way interaction for *d*′ between final test condition (item recognition vs associative recognition), diversity (high vs low), and word frequency (high vs low). For item recognition, performance was best for items that were both low frequency and low diversity but, for associative recognition, performance was best for words that were high frequency and low diversity. They also found a significant three-way interaction between encoding condition (associative vs item), training condition, and diversity. Associative memory for low-diversity items benefitted most from associative encoding and associative training, whereas memory for associations between high-diversity items was significantly attenuated by item encoding and associative training. Cook et al. (2006) found that when words were studied for 2,000 or 500 ms hit rates were higher for low- than high-diversity and for low- than high-frequency words, whereas false-alarm rates were higher for high- than low-diversity and low- than high-frequency words. Marsh et al., (2006a, 2006b) found a three-way interaction between source (heard vs generated), diversity (high vs low), and word frequency (high vs low). Source memory was affected by frequency and diversity for heard items, with source identification being more accurate for items that were low frequency and low diversity, but only by diversity for generated items, with low-diversity items being identified more accurately than high-diversity items. Papesh et al. (2019) found that *d*_a_ was lowest for lures drawn from a high diversity/frequency distribution, whereas *d*_a_ for targets did not significantly differ between conditions.

17 Aka et al. (2021) did not directly control for frequency, but their word pool did not contain words with extreme frequencies.

18 They found no effect of diversity on order errors in either experiment and significantly fewer item errors for low-diversity lists in Experiment 1, but no effect of diversity on item errors in Experiment 2.

19 Lexical decision and word naming tasks were described in the Behavioural Studies section. Word prevalence, a measure of how many individuals know a word, was calculated using a variant of lexical decision (Brysbaert et al., 2019). Participants were asked to indicate whether they ‘knew’ a word or not, and the proportion of people who knew a word and their decision times were recorded. For word familiarity ratings, participants rate on a 7-point scale how familiar they are with a word (Clark & Pavio, 2004). For idiom familiarity, participants rate on a 5-point scale how familiar they are with idiomatic phrases with a verb-determiner-noun structure (Libben & Titone, 2008). Note that these measures do not necessarily require participants to know the meaning of the word or phrase.

20 rank CD; LogCD; PowerCD; generalised power CD; raceCD; APEXCD; IA1CD; IA2CD

21 British National Corpus, CELEX, Kucera-Francis and TASA

22 Excepting user SDM diversity using a word representation for the English Lexicon Project LDT accuracy where word frequency explained slightly more variance.

23 All eye-tracking studies covered here placed words in neutral sentences that did not contain contextual information about their meaning

## Code availability

There is no code associated with this study.

## Declarations

### Author note

Special thanks to Louise Wong for their help with the article selection process and Breege Whiten for their help developing the final search strategy.

### Open science statement

All data and experimental materials are publicly available via the Open Science Framework (https://osf.io/bpgr9/).
